# Interpretable machine learning for postoperative sepsis prediction in ICU patients following intracranial hematoma evacuation

**DOI:** 10.3389/fcimb.2026.1895258

**Published:** 2026-09-22

**Authors:** Shaoyang Yu, Yun Liu, Lei Guo, Jinchang He, Yuyuan Ge, Wan Yu, Weijian Jia, Kexiang Xiong, Zixiang Wang, Wei Gao, Yong Zhang

**Affiliations:** 1 The Second School of Clinical Medicine, Nanjing Medical University, Nanjing, Jiangsu, China; 2 Department of Neurosurgery, Affiliated Hospital of Integrated Traditional Chinese and Western Medicine, Nanjing University of Chinese Medicine, Nanjing, Jiangsu, China; 3 Department of Neurosurgery, Jiangsu Province Academy of Traditional Chinese Medicine, Nanjing, Jiangsu, China; 4 School of Life Sciences, Xiamen University, Xiamen, Fujian, China; 5 Department of Critical Care Medicine, Jiangsu Province Academy of Traditional Chinese Medicine, Nanjing, Jiangsu, China

**Keywords:** machine learning, multi-database validation, postoperative sepsis, SHAP analysis, XGBoost

## Abstract

**Background:**

Sepsis is a prevalent and life-threatening complication in ICU patients after intracranial hematoma evacuation. This study aimed to develop and externally validate an interpretable machine learning model for predicting sepsis risk in this high-risk neurocritical care population.

**Methods:**

A multicenter retrospective study was designed. The MIMIC-IV was based on for model development and internal testing; MIMIC-III, eICU and an independent clinical cohort from Jiangsu Provincial Hospital of Integrated Traditional Chinese and Western Medicine for external validation. Sepsis during ICU stay was defined according to the operationalized Sepsis-3 criteria. Early ICU variables were selected using Boruta and LASSO, and six machine learning algorithms were developed and compared. Model performance was evaluated by discrimination, calibration, and decision curve analysis, and its interpretability was assessed using SHAP and LIME. A sensitivity analysis was conducted by retraining all six models after excluding baseline SOFA.

**Results:**

A total of 1,729 patients were included, of whom 717 developed sepsis during ICU stay. The final model retained 13 predictors, including WBC, SOFA score, SpO_2_, glucose, chloride, pneumonia, aspirin use, mannitol use, vasopressor use, mechanical ventilation, RBC, sedative use, and GCS score. XGBoost showed the most balanced overall performance, with AUCs of 0.794 in the internal test set and 0.820, 0.837, and 0.796 in the MIMIC-III, eICU, and independent clinical validation cohorts, respectively. SHAP analysis identified SOFA score, vasopressor use, glucose, pneumonia, and sedative exposure as major contributors to model predictions, while LIME supported individual-level interpretability. A web-based calculator was implemented for individualized risk prediction. Sensitivity analysis showed that XGBoost retained discrimination after SOFA exclusion, with no significant AUC changes across the external cohorts.

**Conclusion:**

This study developed and externally validated an interpretable XGBoost-based model for predicting sepsis risk during ICU stay among patients after intracranial hematoma evacuation. The model showed generally consistent AUC-based discrimination across multiple validation cohorts and may support individualized risk stratification. Further prospective validation and clinical impact assessment are required before routine clinical implementation.

## Introduction

Surgical intervention remain the first choice for patients with intracranial hematoma, including those with spontaneous intracerebral hemorrhage, traumatic brain injury–associated hemorrhage, and space-occupying intracranial hematoma. In the acute phase, intracranial hematoma evacuation can relieve intracranial pressure, prevent progressive neurological deterioration, and reduce the risk of brain herniation; however, adverse events usually develop during postoperative ICU admission for neurological monitoring and organ support (Valadka and Robertson, 2007; de Oliveira Manoel et al., 2016). Despite advances in neuroimaging, perioperative management, and neurocritical care, these events still lead to a poor prognosis and a high mortality earlier in ICU admission (GBD Traumatic Brain Injury Injury and Spinal Cord Injury Collaborators, 2019; Feigin et al., 2024; Liu et al., 2026).

The Global Burden of Disease Study estimated approximately 48.9 million sepsis cases and 11.0 million sepsis-related deaths worldwide in 2017, accounting for 19.7% of all global deaths (La Via et al., 2024). Sepsis is particularly common in intensive care settings. A global ICU audit reported sepsis in 29.5% of ICU patients, with ICU and hospital mortality rates of 25.8% and 35.3%, respectively (Vincent et al., 2014). Therefore, early identification of sepsis risk in ICU patients may bring a clinical benefit to patients’ safety (Seymour et al., 2017).

In patients receiving intracranial hematoma evacuation, infection and secondary sepsis may result from the combination of pathophysiological and hospitalization-related factors. Acute brain injury may disrupt neuroimmune homeostasis through autonomic and neuroendocrine pathways, triggering early systemic inflammation followed by brain injury–induced immunosuppression and susceptibility to nosocomial infection (Simon et al., 2017; Shi et al., 2018). Impaired consciousness, mechanical ventilation, sedative exposure, circulatory instability, and metabolic disturbances may further increase the risk of hospital-acquired infection and sepsis (Turina et al., 2005; Athar et al., 2012; Singer et al., 2016; Filis et al., 2018; Hsu et al., 2022; Kulikov et al., 2022; Zhou et al., 2022; Howroyd et al., 2024; Monnet et al., 2025). Conventional severity scores, such as SOFA and SAPS II, provide a general assessment of illness severity but were not specifically developed for patients undergoing intracranial hematoma evacuation. In this population, postoperative sepsis risk may also be associated with neurological status, infection burden, physiological disturbances, and treatment exposures (Schertz et al., 2023). Therefore, we sought to evaluate whether integrating routinely available early ICU variables could support individualized postoperative sepsis risk stratification.

Machine learning (ML), which can integrate multidimensional ICU data and capture complex nonlinear relationships, has been increasingly adopted to predict sepsis risk in neurocritical care populations (Liu et al., 2024; Liu et al., 2025; Wu et al., 2025). Explainability methods such as SHAP and LIME can ensure transparency and clinical interpretability at both global and individual levels (Rodriguez-Perez and Bajorath, 2020). However, existing studies mainly focus on general ICU populations or specific disease subgroups, such as isolated intracerebral hemorrhage or traumatic brain injury (Zhou et al., 2026). In postoperative or perioperative settings, most of related studies have included broadly defined surgical populations (Zhang et al., 2025). Moreover, the lack of independent external validation across heterogeneous databases leaves their generalizability uncertain in patients after intracranial hematoma evacuation. Patients undergoing intracranial hematoma evacuation represent a specific postoperative neurocritical care population in whom acute neurological injury and postoperative exposures, including airway management, mechanical ventilation, sedation, and circulatory support, may jointly influence the risk of infection and subsequent sepsis.

To address these gaps, we developed and externally validated an interpretable ML model for predicting sepsis risk in ICU patients after intracranial hematoma evacuation. MIMIC-IV was used for model development and internal testing, while MIMIC-III, eICU, and an independent clinical cohort from Jiangsu Provincial Hospital of Integrated Traditional Chinese and Western Medicine were used for external validation. Multiple supervised ML algorithms were compared, and the best-performing model was further interpreted using SHAP and LIME, and deployed as a publicly accessible web-based prediction tool to support individualized risk stratification.

## Materials and methods

### Data sources and study design

This multicenter retrospective cohort study aimed to develop and validate machine learning models for predicting sepsis risk in ICU patients after intracranial hematoma evacuation using multiple critical care databases and an independent clinical validation cohort. Model development was performed using MIMIC-IV, with external validation conducted in MIMIC-III, eICU, and an independent clinical cohort from Jiangsu Provincial Hospital of Integrated Traditional Chinese and Western Medicine to assess robustness and generalizability across different temporal, healthcare, and real-world clinical settings. MIMIC-IV, MIMIC-III, and eICU are de-identified publicly available databases; therefore, additional informed consent was not required. One of the authors (YSY) obtained access to the data-base (certification number: 76647351). The study protocol for the institutional validation cohort was approved by the ethics committee of Jiangsu Provincial Hospital of Integrated Traditional Chinese and Western Medicine, with informed consent waived owing to the retrospective study design(approval number: 2026-LWKYZ-015).**Figure 1** provides a structured overview of the study design and analytical workflow, while the individual components are described in detail in the corresponding Methods and Results sections.

**Figure 1:**
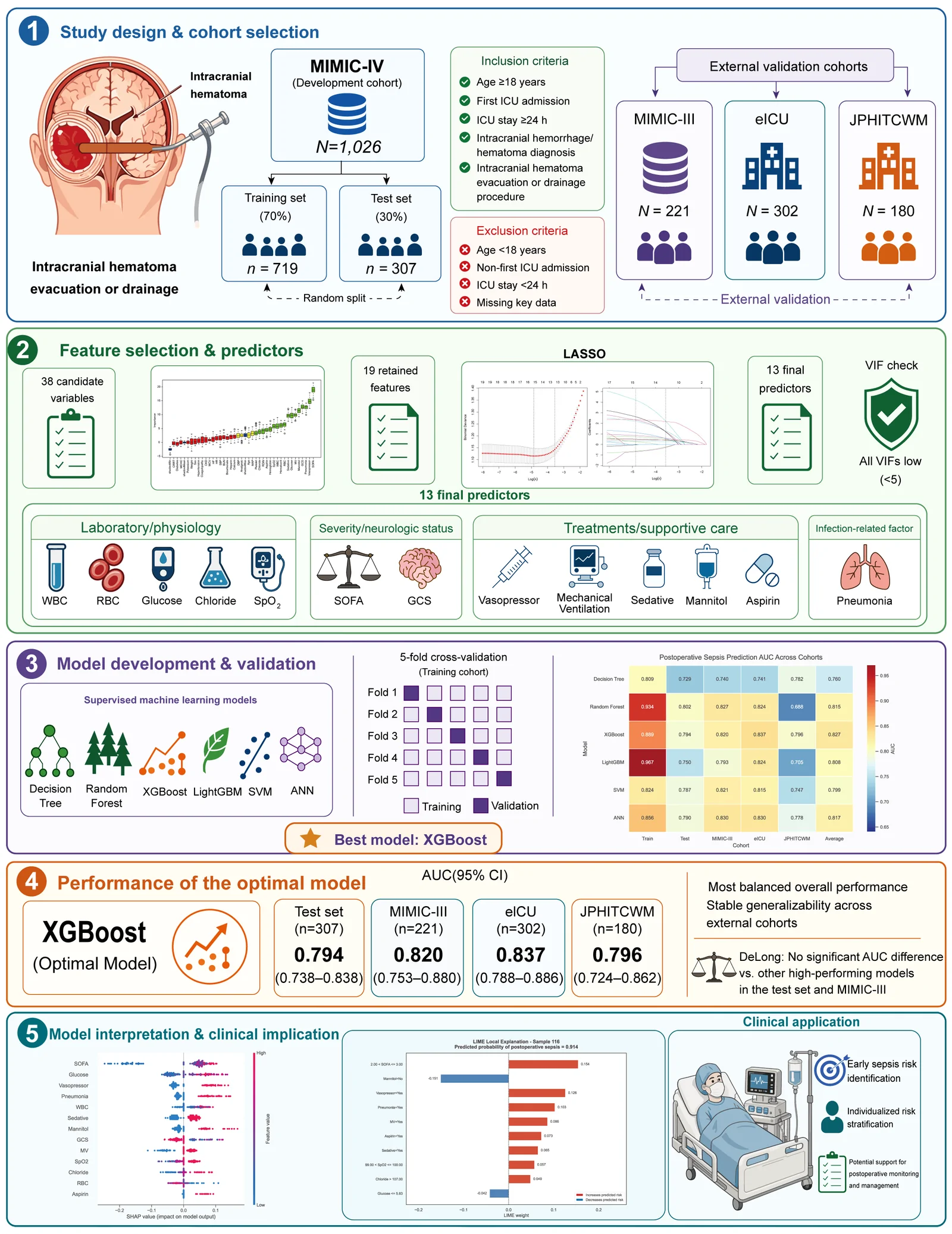
Overview of the study design, analytical workflow, and key findings. The figure provides a structured overview of cohort selection and allocation, external validation using the MIMIC-III, eICU, and JPHITCWM cohorts, feature selection with Boruta and LASSO, development and validation of six machine learning models, and interpretation of the final model. Considering its overall discrimination, calibration, and decision-curve net benefit across the evaluated cohorts, XGBoost was selected as the primary model for postoperative sepsis risk prediction. The potential clinical application of the model for early risk identification, individualized risk stratification, and postoperative monitoring is also illustrated.

### Database descriptions

#### MIMIC-IV database

Data were extracted from the Medical Information Mart for Intensive Care IV (MIMIC-IV) database, which contains de-identified clinical data of ICU patients admitted to Beth Israel Deaconess Medical Center in the United States between 2008 and 2019 (Johnson et al., 2023). MIMIC-IV served as the primary development dataset for model construction and internal testing and was randomly divided into training and test sets at a ratio of 7:3.

#### MIMIC-III database

Temporal external validation was performed using the Medical Information Mart for Intensive Care III (MIMIC-III) database (Goldberger et al., 2000), which contains de-identified clinical data of ICU patients from the same institution between 2001 and 2012. In this study, MIMIC-III was used as an external validation cohort to assess model stability in patient populations from a different time period.

#### eICU database

Geographical external validation was further performed using the eICU Collaborative Research Database (eICU-CRD) (Pollard et al., 2018), which contains multicenter de-identified clinical data from 335 ICUs across 208 hospitals in the United States between 2014 and 2015. In this study, eICU was used to evaluate the generalizability of the model across different healthcare institutions and clinical settings.

#### JPHITCWM database

The fourth validation dataset was an independent clinical cohort from Jiangsu Provincial Hospital of Integrated Traditional Chinese and Western Medicine. It included adult ICU patients hospitalized after intracranial hematoma evacuation between January 2022 and April 2026. The independent JPHITCWM cohort included demographic, clinical, treatment, and outcome data during the ICU stay and was used to further assess model generalizability and robustness in real-world clinical practice.

#### Study population and eligibility criteria

The study cohort was identified using a combined diagnosis- and procedure-code strategy. Because the MIMIC databases do not contain a single standardized procedure code that specifically identifies “intracranial hematoma evacuation,” patients were required to have both ICD diagnosis codes for intracranial hemorrhage or hematoma and procedure codes for intracranial hematoma evacuation or related neurosurgical procedures during the same hospitalization. This strategy was intended to reduce the inclusion of conservatively treated hemorrhage cases and patients who underwent neurosurgical procedures for unrelated conditions.

Diagnostic codes included ICD-10 codes beginning with S06.4, S06.5, S06.3, I61, and I62, and ICD-9 codes 431, 432.0, 432.1, 432.9, 852.2–852.5, 853, and 851. Procedure codes included ICD-10-PCS codes 00C0–00C4, 0090, 0091, 0092, 0093, and 0094, and ICD-9 procedure codes 01.24, 01.26, 01.28, 01.31, 01.09, and 01.39. Only patients meeting both diagnostic and procedure-code criteria were included. This combined approach was used to improve case-identification specificity and reduce potential misclassification.

Inclusion criteria were age ≥18 years, first ICU admission during the hospitalization, ICU length of stay ≥24 h, diagnosis of intracranial hemorrhage or hematoma, and receipt of intracranial hematoma evacuation. Exclusion criteria were substantial missingness in key clinical variables, non-first ICU admission. This combined coding strategy, together with restrictions on ICU admission and length of stay, was used to improve cohort specificity and clinical comparability.

#### Data extraction and processing

Demographic data, vital signs, laboratory results, and clinical outcomes were extracted from the structured tables of MIMIC-IV, MIMIC-III, and eICU using PostgreSQL queries. For the JPHITCWM cohort, clinical and laboratory variables were extracted from the electronic medical records of all eligible patients. All candidate predictors were defined using the first available values recorded after ICU admission and before sepsis diagnosis. A total of 38 candidate features were collected, covering demographics, vital signs, laboratory tests, severity scores, interventions, and comorbidities. Variable definitions were harmonized across data sources as far as possible using structured records and International Classification of Diseases (ICD) codes. Continuous variables were not standardized or normalized before modeling. Sample size adequacy was assessed according to the principle of at least 10 events per variable (EPV), to ensure model stability and reliability.

#### Outcome definition

The primary outcome was sepsis occurring during the ICU stay, defined according to the Sepsis-3 criteria (Singer et al., 2016). Specifically, sepsis was defined as suspected or confirmed infection accompanied by an acute increase in the Sequential Organ Failure Assessment (SOFA) score of ≥2 points. The outcome variable was treated as binary (yes/no).

#### Statistical analysis

Statistical analyses were performed using R version 4.5.1 and Python version 3.10.5. Variables with <20% missingness were imputed using the mice package with five imputations (m = 5; seed = 123), whereas variables with higher missingness were excluded. Missingness was summarized using missing-data plots and missingness proportion tables. The MIMIC-IV cohort was randomly divided into training and test sets at a 7:3 ratio using outcome-stratified sampling with caret::createDataPartition (seed = 123). Baseline characteristics were summarized using the tableone package. Continuous variables were assessed for normality using the Shapiro–Wilk test and reported as mean ± standard deviation or median (interquartile range), as appropriate. Categorical variables were reported as frequencies and percentages. All tests were two-sided, and P < 0.05 was considered statistically significant.

Feature selection was performed exclusively in the MIMIC-IV training set to avoid information leakage. Boruta was first used for broad screening of potentially relevant variables because its random forest–based comparison with shadow features can accommodate nonlinear relationships, followed by LASSO regression with ten-fold cross-validation for regularization and further dimensionality reduction to obtain a parsimonious predictor set. Variance inflation factors (VIFs) were calculated to assess multicollinearity among the candidate predictors. Pairwise correlations among the 13 predictors retained in the final models were subsequently evaluated using Spearman correlation coefficients, with |r_s_| ≥ 0.70 considered indicative of strong correlation.

Six supervised machine learning models were developed: decision tree, random forest (RF), extreme gradient boosting (XGBoost), LightGBM, support vector machine (SVM), and artificial neural network (ANN). Hyperparameters were optimized exclusively within the training set using grid search with five-fold cross-validation, with the mean cross-validated AUC as the optimization criterion. The parameter combination achieving the highest mean AUC was used to refit each model on the complete training set. The optimized models were subsequently evaluated in the internal test set and three external validation cohorts without further tuning or updating.

Model performance was assessed in the training set, test set, and external validation cohorts. Discrimination was evaluated using ROC curves and AUCs, with 95% confidence intervals estimated using 1,000 bootstrap resamples. Additional metrics included accuracy, precision/positive predictive value, sensitivity/recall, specificity, F1 score, Cohen’s kappa, Youden’s J statistic, and negative predictive value. Calibration was assessed using calibration curves based on 10 equally spaced probability bins and Brier scores, with 95% confidence intervals for the Brier scores estimated using 1,000 bootstrap resamples. Clinical utility was evaluated using decision curve analysis across threshold probabilities from 0.01 to 0.99, with comparison against the treat-all and treat-none strategies. DeLong tests were used for pairwise comparisons of correlated ROC AUCs among candidate models. The final model was selected based on its overall performance and consistency across the internal and external validation cohorts, considering discrimination, calibration, classification metrics, and decision-curve net benefit rather than performance in a single cohort.

The final model was interpreted using Shapley additive explanations (SHAP) and local interpretable model-agnostic explanations (LIME). SHAP was used to assess global and individual-level feature contributions using feature importance, bees warm, dependence, waterfall, and force plots. LIME was applied to representative individual cases to evaluate local feature contributions to model predictions. Together, SHAP and LIME provided complementary global and local explanations, improving model transparency and clinical interpretability.

#### Sensitivity analysis

To evaluate whether predictive performance was predominantly driven by baseline disease severity or overlapping information from the composite SOFA score, a sensitivity analysis was conducted. All six machine learning models were retrained after excluding baseline SOFA while retaining the remaining 12 predictors. The restricted models were evaluated using the same model-development and validation framework as in the primary analysis. Their performance was compared with that of the original models, and differences in AUC were assessed using paired DeLong tests in the internal test set and the three external validation cohorts. SHAP analysis was additionally performed for the SOFA-free XGBoost model to examine the redistribution of feature contributions after SOFA exclusion.

## Results

### Patient selection and baseline characteristics

**Figure 1** summarizes the study design, cohort allocation, and analytical framework. After systematic screening, 1,729 ICU patients after intracranial hematoma evacuation were included. MIMIC-IV included 1,026 patients and was divided into a training set (n=719) and an internal validation set (n=307) at a 7:3 ratio. MIMIC-III, eICU, and JPHITCWM included 221, 302, and 180 patients, respectively, and served as external validation cohorts. The main exclusion criteria were non-first ICU admission, age <18 years, ICU length of stay <24 h, absence of procedure records related to intracranial hematoma evacuation, absence of corresponding intracranial hemorrhage or hematoma diagnoses, and missing key clinical information. Missingness patterns are shown in **Supplementary Figures 1**-**4**, and the missingness rates of candidate variables included in the final analysis were generally acceptable.

Baseline characteristics of the four cohorts are shown in **Table 1**. The overall median age was 69.0 years (IQR, 57.0–79.0), and 65.9% of patients were male. Postoperative sepsis occurred in 717 patients, with an overall incidence of 41.5%. The incidence differed significantly across cohorts, with 50.5% in MIMIC-IV, 29.4% in MIMIC-III, 25.5% in eICU, and 31.7% in JPHITCWM (P<0.001), potentially reflecting differences in case mix, disease severity, and clinical management. Clinical characteristics, including age, body weight, SOFA score, GCS score, blood pressure, SpO_2_, treatment exposures, and comorbidities, also varied across cohorts. The JPHITCWM cohort was relatively younger but had a substantially higher median baseline SOFA score than MIMIC-IV (7 [IQR, 6–10] vs. 3 [IQR, 2–4]; P < 0.001), together with lower GCS scores and more frequent use of organ-supportive treatments, indicating an important severity-related case-mix difference.

**Table 1:** Comparison of baseline characteristics of ICU patients after intracranial hematoma evacuation across four databases.

Characteristics	Level	Overall(n=1729)	MIMIC-IV(n=1026)	MIMIC-III(n=221)	eICU (n=302)	JPHITCWM (n=180)	*p*
Sepsis during ICU stay	Nonsepsis	1012 (58.53)	508 (49.51)	156 (70.59)	225 (74.50)	123 (68.33)	<0.001
	Sepsis	717 (41.47)	518 (50.49)	65 (29.41)	77 (25.50)	57 (31.67)	
Sex	Female	589 (34.07)	346 (33.72)	75 (33.94)	110 (36.42)	58 (32.22)	0.783
	Male	1140 (65.93)	680 (66.28)	146 (66.06)	192 (63.58)	122 (67.78)	
MV	No	587 (33.95)	274 (26.71)	106 (47.96)	193 (63.91)	14 (7.78)	<0.001
	Yes	1142 (66.05)	752 (73.29)	115 (52.04)	109 (36.09)	166 (92.22)	
Vasopressor	No	1296 (74.96)	793 (77.29)	169 (76.47)	248 (82.12)	86 (47.78)	<0.001
	Yes	433 (25.04)	233 (22.71)	52 (23.53)	54 (17.88)	94 (52.22)	
CRRT	No	1712 (99.02)	1014 (98.83)	221 (100.00)	298 (98.68)	179 (99.44)	0.353
	Yes	17 (0.98)	12 (1.17)	0 (0.00)	4 (1.32)	1 (0.56)	
Mannitol	No	1402 (81.09)	881 (85.87)	169 (76.47)	290 (96.03)	62 (34.44)	<0.001
	Yes	327 (18.91)	145 (14.13)	52 (23.53)	12 (3.97)	118 (65.56)	
Aspirin	No	1651 (95.49)	965 (94.05)	215 (97.29)	292 (96.69)	179 (99.44)	0.003
	Yes	78 (4.51)	61 (5.95)	6 (2.71)	10 (3.31)	1 (0.56)	
Heparin	No	550 (31.81)	106 (10.33)	24 (10.86)	243 (80.46)	177 (98.33)	<0.001
	Yes	1179 (68.19)	920 (89.67)	197 (89.14)	59 (19.54)	3 (1.67)	
Sedative	No	779 (45.05)	534 (52.05)	73 (33.03)	156 (51.66)	16 (8.89)	<0.001
	Yes	950 (54.95)	492 (47.95)	148 (66.97)	146 (48.34)	164 (91.11)	
Analgesic	No	148 (8.56)	42 (4.09)	2 (0.90)	65 (21.52)	39 (21.67)	<0.001
	Yes	1581 (91.44)	984 (95.91)	219 (99.10)	237 (78.48)	141 (78.33)	
Hypertension	No	856 (49.51)	461 (44.93)	92 (41.63)	217 (71.85)	86 (47.78)	<0.001
	Yes	873 (50.49)	565 (55.07)	129 (58.37)	85 (28.15)	94 (52.22)	
HF	No	1592 (92.08)	927 (90.35)	196 (88.69)	295 (97.68)	174 (96.67)	<0.001
	Yes	137 (7.92)	99 (9.65)	25 (11.31)	7 (2.32)	6 (3.33)	
Diabetes	No	1417 (81.95)	783 (76.32)	215 (97.29)	280 (92.72)	139 (77.22)	<0.001
	Yes	312 (18.05)	243 (23.68)	6 (2.71)	22 (7.28)	41 (22.78)	
CKD	No	1603 (92.71)	931 (90.74)	211 (95.48)	288 (95.36)	173 (96.11)	0.002
	Yes	126 (7.29)	95 (9.26)	10 (4.52)	14 (4.64)	7 (3.89)	
Pneumonia	No	1438 (83.17)	849 (82.75)	175 (79.19)	287 (95.03)	127 (70.56)	<0.001
	Yes	291 (16.83)	177 (17.25)	46 (20.81)	15 (4.97)	53 (29.44)	
Coagulopathy	No	1465 (84.73)	821 (80.02)	179 (81.00)	285 (94.37)	180 (100.00)	<0.001
	Yes	264 (15.27)	205 (19.98)	42 (19.00)	17 (5.63)	0 (0.00)	
Age(years)(median [IQR])		69.00 [57.00, 79.00]	70.00 [59.00, 80.00]	69.00 [57.00, 79.00]	71.00 [57.00, 81.00]	58.00 [49.00, 69.25]	<0.001
Weight(kg) (median [IQR])		75.00 [64.60, 86.60]	75.32 [63.90, 87.10]	77.20 [68.00, 89.80]	74.90 [66.03, 88.40]	70.00 [62.00, 80.00]	<0.001
SOFA (median [IQR])		3.00 [2.00, 5.00]	3.00 [2.00, 4.00]	3.00 [2.00, 4.00]	3.00 [1.00, 5.00]	7.00 [6.00, 10.00]	<0.001
GCS (median [IQR])		14.00 [10.00, 15.00]	14.00 [12.00, 15.00]	14.00 [11.00, 15.00]	14.00 [10.00, 15.00]	3.00 [2.00, 8.00]	<0.001
HR(bpm)(median [IQR])		78.00 [68.00, 89.00]	77.00 [67.00, 89.00]	78.00 [69.00, 89.00]	78.00 [68.00, 88.75]	80.00 [69.75, 90.00]	0.316
SBP(mmHg)(median [IQR])		131.00 [117.00, 146.00]	129.00 [115.00, 144.00]	134.00 [117.00, 145.00]	134.00 [122.00, 148.00]	135.00 [115.75, 158.00]	<0.001
DBP(mmHg)(median [IQR])		70.00 [60.00, 81.00]	70.00 [61.00, 82.00]	69.00 [59.00, 80.00]	71.00 [60.00, 79.00]	71.00 [61.75, 81.00]	0.849
MAP(mmHg)(median [IQR])		86.70 [76.00, 97.00]	85.00 [76.00, 97.00]	85.00 [73.00, 95.00]	88.00 [78.00, 97.00]	93.68 [82.17, 103.75]	<0.001
SpO^2^(%)(median [IQR])		99.00 [97.00, 100.00]	99.00 [97.00, 100.00]	99.00 [97.00, 100.00]	99.00 [97.00, 100.00]	100.00 [99.00, 100.00]	<0.001
Glucose(mmol/L)(median [IQR])		7.39 [6.11, 9.39]	6.94 [5.78, 8.72]	7.11 [6.11, 8.67]	8.47 [7.00, 10.54]	9.20 [7.19, 11.31]	<0.001
Chloride(mmol/L)(median [IQR])		105.00 [102.00, 109.00]	104.00 [101.00, 107.00]	105.00 [102.00, 108.00]	109.00 [105.00, 113.00]	107.40 [104.30, 111.00]	<0.001
Potassium(mmol/L)(median [IQR])		4.00 [3.70, 4.30]	3.90 [3.60, 4.30]	3.90 [3.60, 4.20]	4.30 [4.00, 4.60]	3.78 [3.43, 4.16]	<0.001
BUN(mg/dL)(median [IQR])		15.12 [11.00, 21.00]	14.00 [11.00, 19.00]	15.00 [11.00, 19.00]	20.00 [14.00, 29.75]	17.40 [13.42, 24.86]	<0.001
Hematocrit(%)(median [IQR])		34.60 [30.40, 38.50]	34.70 [30.40, 38.30]	33.50 [29.90, 38.30]	35.70 [32.15, 39.92]	32.65 [28.55, 37.62]	<0.001
Hemoglobin(g/dL)(mean (SD))		11.51 (2.08)	11.46 (2.03)	11.53 (2.01)	12.03 (1.93)	10.85 (2.49)	<0.001
RDW(%)(median [IQR])		13.70 [13.00, 15.00]	13.60 [12.90, 14.80]	13.90 [13.30, 15.10]	14.50 [13.60, 16.00]	13.20 [12.60, 13.90]	<0.001
Platelet(×10^9^/L)(median [IQR])		206.00 [157.00, 265.00]	202.00 [156.25, 255.00]	209.00 [159.00, 262.00]	261.00 [205.00, 337.50]	157.00 [111.00, 196.50]	<0.001
WBC(×10^9^/L)(median [IQR])		10.00 [7.60, 13.30]	9.20 [6.90, 12.10]	8.90 [6.70, 11.40]	12.80 [9.80, 16.38]	12.31 [9.52, 15.98]	<0.001
RBC(×10¹²/L)(mean (SD))		3.80 (0.71)	3.80 (0.70)	3.83 (0.71)	4.00 (0.64)	3.47 (0.81)	<0.001
Bicarbonate(mmol/L)(median [IQR])		24.00 [22.00, 26.00]	23.00 [21.00, 25.00]	25.00 [23.00, 27.00]	27.00 [25.00, 29.00]	22.70 [20.80, 24.90]	<0.001
Calcium(mg/dL)(median [IQR])		8.62 [8.20, 9.10]	8.65 [8.20, 9.10]	8.60 [8.20, 9.10]	8.90 [8.60, 9.30]	8.10 [7.65, 8.46]	<0.001
MCHC(g/dL)(median [IQR])		33.60 [32.60, 34.40]	33.20 [32.40, 34.20]	34.00 [33.00, 35.00]	34.00 [33.10, 34.70]	33.70 [33.10, 34.23]	<0.001
MCV(fL)(median [IQR])		92.00 [87.00, 95.00]	92.00 [87.00, 95.00]	90.00 [85.00, 94.00]	92.00 [87.93, 95.42]	92.45 [89.40, 94.50]	<0.001

### Feature selection and variable importance ranking

A total of 38 candidate predictors, excluding the outcome variable, were included in this study, covering demographics, therapeutic interventions such as mechanical ventilation, vasopressor use, and sedative therapy, comorbidities, vital signs, laboratory tests, and disease severity scores.

To identify the predictors most relevant to the outcome, a two-step feature selection strategy was applied. First, the Boruta algorithm, a random forest–based wrapper feature selection method, was used to screen potentially relevant variables, resulting in 19 predictors retained for further analysis (**Figure 2A**). Subsequently, least absolute shrinkage and selection operator (LASSO) regression was applied to the Boruta-selected variables for dimensionality reduction and shrinkage, with the aim of reducing redundant variables and mitigating potential multicollinearity. After the optimal penalty parameter λ was determined by ten-fold cross-validation, 13 key predictors were finally selected for model development: WBC, SOFA score, SpO_2_, glucose, chloride, pneumonia, aspirin use, mannitol use, vasopressor use, mechanical ventilation, RBC, sedative use, and GCS score (**Figures 2B, C**).

**Figure 2:**
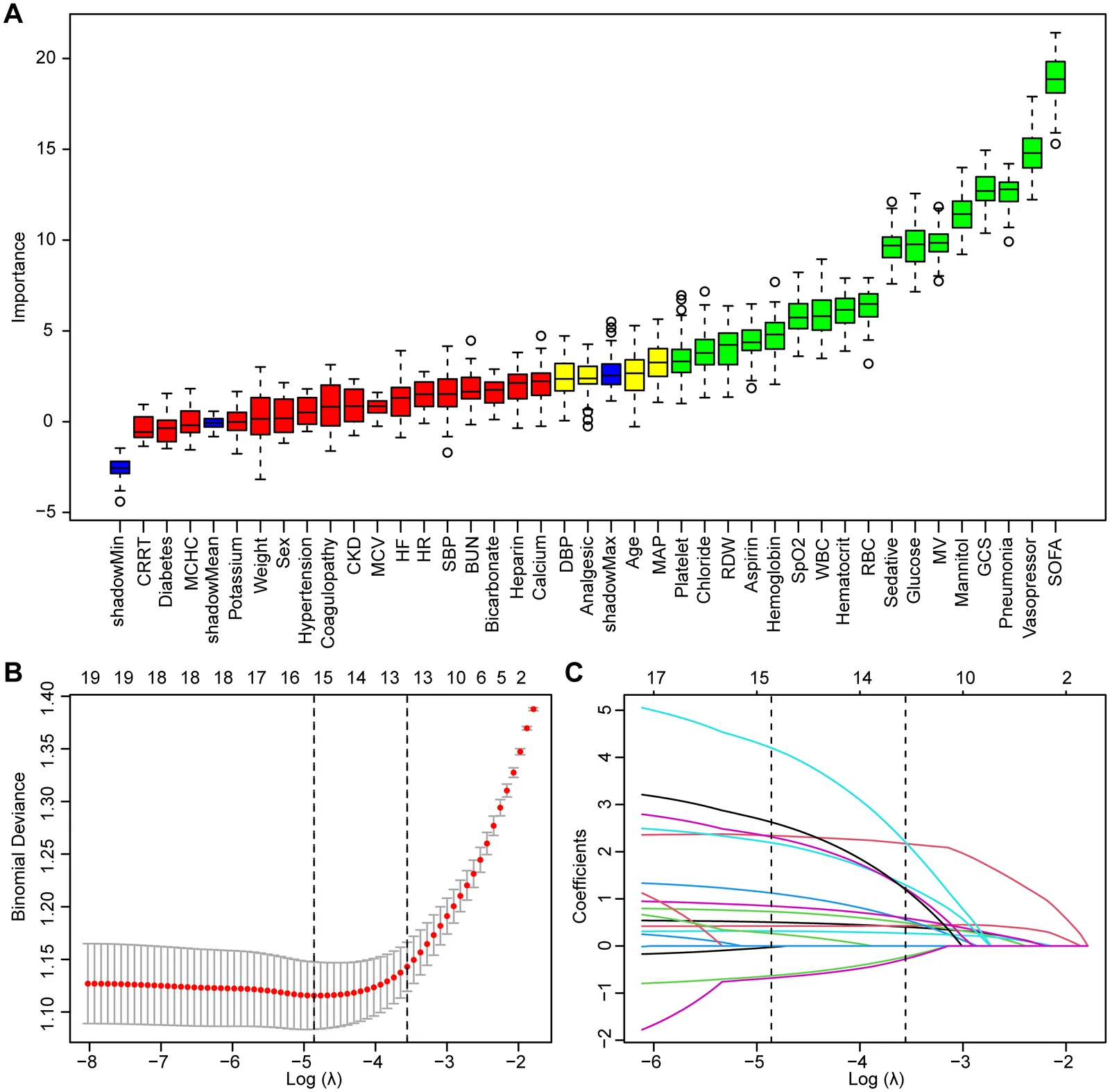
Feature selection process and final predictor selection. **(A)** Feature importance identified by the Boruta algorithm (green: confirmed, red:rejected, yellow: tentative). **(B)** Cross-validation curve used to determine the optimal penalty parameter in LASSO. Thirteen predictors were ultimately retained at the λ value that minimized binomial deviance in 10-fold cross-validation. **(C)** Coefficient paths of LASSO regression across varying log(lambda) values.

Collinearity assessment showed that the variance inflation factors of the 13 predictors were close to 1, ranging from approximately 1.01 to 1.54, with no evidence of substantial multicollinearity (**Supplementary Table 1**). Moreover, no pair of final predictors showed a strong Spearman correlation (|r_s_| ≥ 0.70). The strongest correlation involving SOFA was observed with GCS (r_s_ = −0.53), whereas its correlation with vasopressor administration was weaker (r_s_ = 0.30; **Supplementary Figure 25**). Model-specific feature importance analyses showed that SOFA, GCS, SpO_2_, glucose, and chloride made substantial contributions to model predictions across multiple algorithms. Vasopressor administration, pneumonia, mechanical ventilation, and sedative use also showed relatively high importance in some models (**Figure 3**).

**Figure 3:**
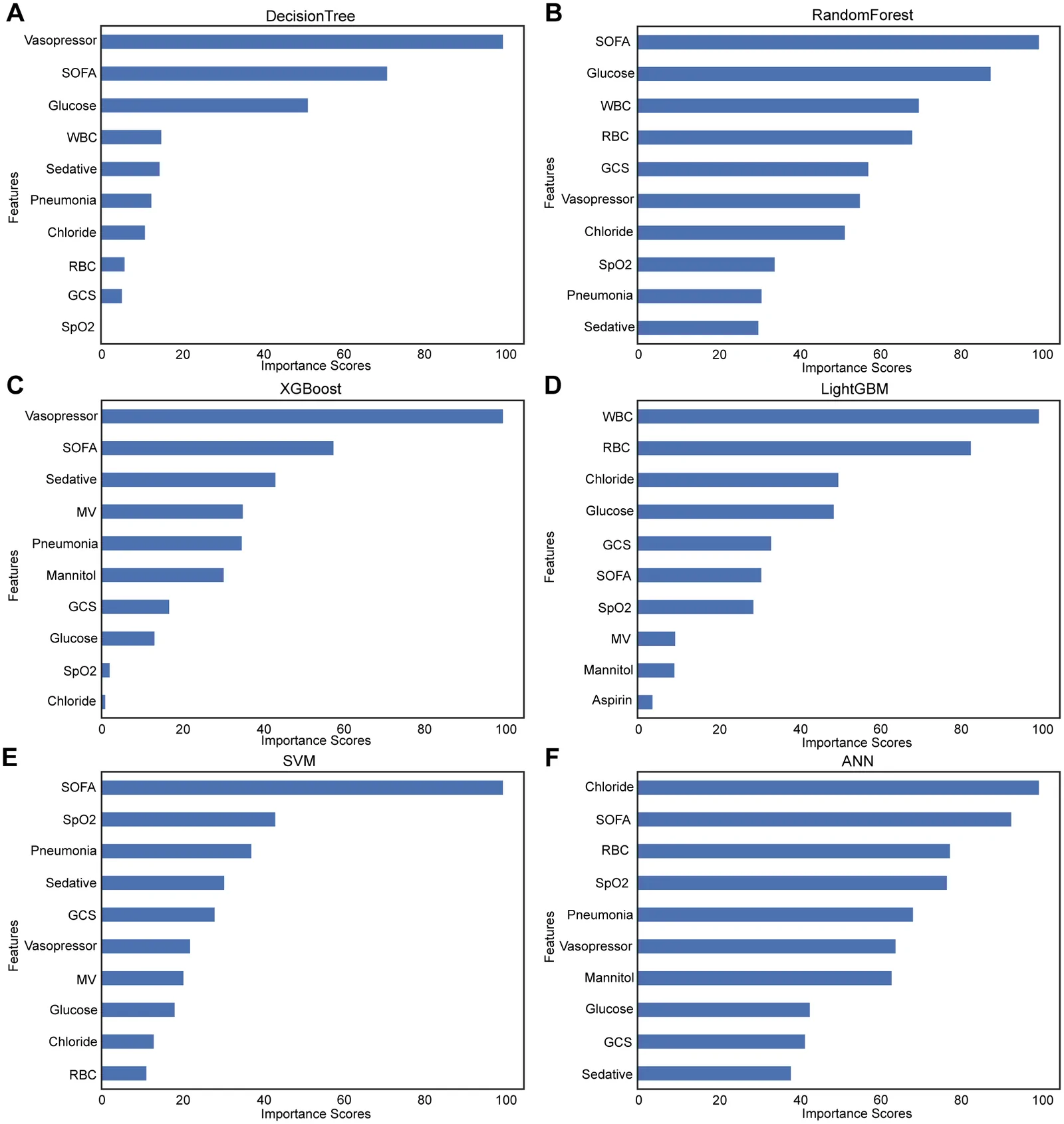
Model-specific feature-importance rankings across six machine learning models: **(A)** decision tree, **(B)** random forest, **(C)** XGBoost, **(D)** LightGBM, **(E)** support vector machine, and **(F)** artificial neural network. Top-ranked predictors were identified using model-specific importance metrics. SOFA score, GCS score, SpO₂, glucose, and chloride were repeatedly identified as important predictors across multiple models. Vasopressor use, pneumonia, mechanical ventilation, and sedative use also showed relatively high importance in some models.

### Model performance comparison

The selected hyperparameters for the six models are summarized in **Supplementary Table 9**.Six machine learning models—decision tree, random forest (RF), XGBoost, LightGBM, support vector machine (SVM), and artificial neural network (ANN)—were compared across the training set, testing set, and three external validation cohorts. Model performance was evaluated in terms of discrimination, calibration, classification performance, and clinical net benefit, including AUC with 95% CI, Brier score, accuracy, sensitivity, specificity, F1 score, Cohen’s kappa, Youden’s index, PPV, NPV, and decision curve analysis. Detailed performance metrics for all models and datasets are summarized in **Table 2**. ROC curves and AUC distributions are presented in **Figure 4**.

**Table 2:** Comprehensive performance metrics of machine learning models for predicting postoperative sepsis after intracranial hematoma evacuation across all datasets.

Data set	Model	AUC	AUC 95% CI lower	AUC 95% CI upper	Accuracy	Precision	Sensitivity	Specificity	F1 score	Kappa	Youden’s J	PPV	NPV
MIMIC IV Train	Decision Tree	0.809	0.780	0.838	0.737	0.708	0.815	0.657	0.758	0.473	0.473	0.708	0.777
	Random Forest	0.934	0.918	0.949	0.846	0.821	0.887	0.803	0.853	0.691	0.690	0.821	0.875
	XGBoost	0.889	0.867	0.910	0.805	0.793	0.832	0.778	0.812	0.610	0.610	0.793	0.820
	LightGBM	0.967	0.956	0.977	0.900	0.896	0.906	0.893	0.901	0.800	0.800	0.896	0.903
	SVM	0.824	0.795	0.852	0.744	0.754	0.733	0.756	0.743	0.488	0.488	0.754	0.735
	ANN	0.856	0.829	0.879	0.771	0.786	0.749	0.792	0.767	0.541	0.541	0.786	0.756
MIMIC IV Test	Decision Tree	0.729	0.671	0.781	0.684	0.671	0.735	0.632	0.702	0.367	0.367	0.671	0.701
	Random Forest	0.802	0.747	0.844	0.713	0.716	0.716	0.711	0.716	0.427	0.427	0.716	0.711
	XGBoost	0.794	0.738	0.838	0.720	0.732	0.703	0.737	0.717	0.440	0.440	0.732	0.709
	LightGBM	0.750	0.687	0.801	0.661	0.662	0.671	0.651	0.667	0.322	0.322	0.662	0.660
	SVM	0.787	0.732	0.833	0.717	0.766	0.632	0.803	0.693	0.434	0.435	0.766	0.682
	ANN	0.790	0.736	0.834	0.710	0.758	0.626	0.796	0.686	0.421	0.422	0.758	0.676
MIMIC-III	Decision Tree	0.740	0.668	0.806	0.593	0.413	0.908	0.462	0.567	0.274	0.369	0.413	0.923
	Random Forest	0.827	0.766	0.883	0.624	0.430	0.846	0.532	0.570	0.295	0.378	0.430	0.892
	XGBoost	0.820	0.753	0.880	0.647	0.449	0.877	0.551	0.594	0.335	0.428	0.449	0.915
	LightGBM	0.793	0.718	0.856	0.643	0.444	0.846	0.558	0.582	0.319	0.404	0.444	0.897
	SVM	0.821	0.761	0.877	0.697	0.491	0.846	0.635	0.621	0.397	0.481	0.491	0.908
	ANN	0.830	0.767	0.883	0.697	0.491	0.815	0.647	0.613	0.388	0.463	0.491	0.894
eICU	Decision Tree	0.741	0.685	0.795	0.586	0.372	0.909	0.476	0.528	0.261	0.385	0.372	0.939
	Random Forest	0.824	0.775	0.874	0.682	0.439	0.896	0.609	0.590	0.376	0.505	0.439	0.945
	XGBoost	0.837	0.788	0.886	0.682	0.437	0.857	0.622	0.579	0.364	0.479	0.437	0.927
	LightGBM	0.824	0.774	0.871	0.699	0.451	0.844	0.649	0.588	0.383	0.493	0.451	0.924
	SVM	0.815	0.761	0.869	0.662	0.417	0.818	0.609	0.553	0.325	0.427	0.417	0.907
	ANN	0.830	0.778	0.879	0.719	0.471	0.831	0.680	0.601	0.408	0.511	0.471	0.922
JPHITCWM	Decision Tree	0.782	0.715	0.842	0.333	0.322	1.000	0.024	0.487	0.016	0.024	0.322	1.000
	Random Forest	0.688	0.608	0.768	0.333	0.322	1.000	0.024	0.487	0.016	0.024	0.322	1.000
	XGBoost	0.796	0.724	0.862	0.328	0.320	1.000	0.016	0.485	0.010	0.016	0.320	1.000
	LightGBM	0.705	0.626	0.783	0.344	0.326	1.000	0.041	0.491	0.026	0.041	0.326	1.000
	SVM	0.747	0.668	0.820	0.339	0.324	1.000	0.033	0.489	0.021	0.033	0.324	1.000
	ANN	0.778	0.713	0.845	0.350	0.328	1.000	0.049	0.494	0.031	0.049	0.328	1.000

**Figure 4:**
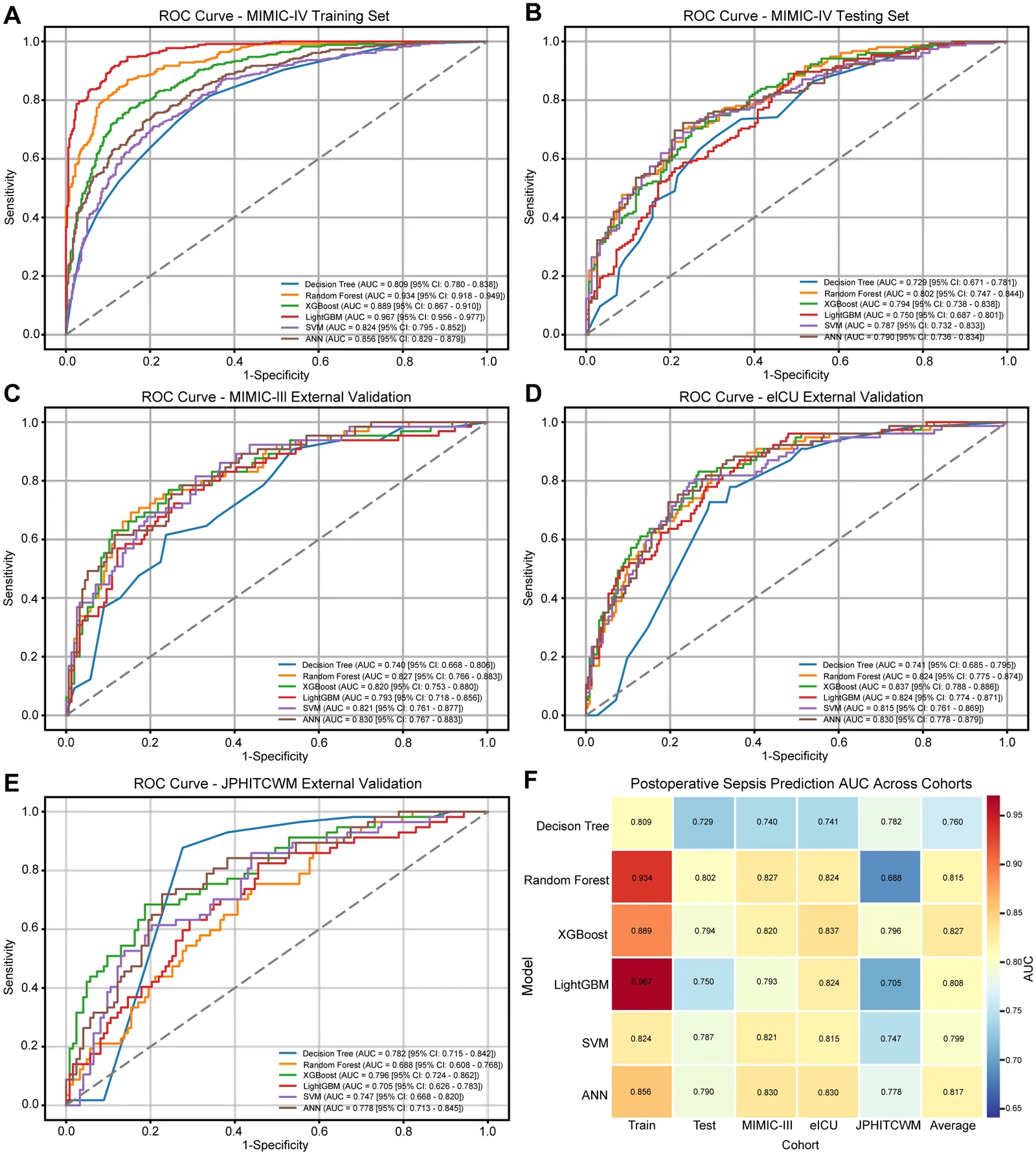
ROC curve analysis and performance heatmap for postoperative sepsis prediction models. **(A–E)** ROC curves comparing six machine learning models across different cohorts, with AUC values displayed in the legends. Models were evaluated in the MIMIC-IV training cohort, MIMIC-IV testing cohort, and three external validation cohorts. **(F)** Heatmap showing AUC values across all models and cohorts, with color intensity representing model performance. **(A)** MIMIC-IV training cohort; **(B)** MIMIC-IV testing cohort; **(C)** MIMIC-III external validation cohort; **(D)** eICU external validation cohort; **(E)** JPHITCWM external validation cohort; **(F)** performance heatmap across all cohorts.

In the training set, LightGBM and RF showed high apparent discrimination, with AUCs of 0.967 (95% CI, 0.956–0.977) and 0.934 (95% CI, 0.918–0.949), respectively. XGBoost also performed well, with an AUC of 0.889 (95% CI, 0.867–0.910), accuracy of 0.805, sensitivity of 0.832, specificity of 0.778, F1 score of 0.812, and Brier score of 0.142 (**Supplementary Figure 5**). However, LightGBM and RF performed substantially better in the training set than in the test and external validation cohorts, suggesting possible overfitting or limited cross-cohort generalizability.

In the test set, model discrimination was generally similar. RF achieved the highest AUC of 0.802 (95% CI, 0.747–0.844), whereas XGBoost achieved a comparable AUC of 0.794 (95% CI, 0.738–0.838). XGBoost maintained balanced classification performance, with accuracy, sensitivity, specificity, and F1 score of 0.720, 0.703, 0.737, and 0.717, respectively. Calibration analysis showed that RF had the lowest Brier score of 0.183, while XGBoost showed a comparable Brier score of 0.186 (**Supplementary Figure 6**). Although RF showed a slight advantage in the test set, XGBoost achieved comparable and balanced performance in discrimination, classification, and calibration, supporting its further evaluation and overall model selection.

In the external validation cohorts, overall discrimination remained acceptable. Because event rates were relatively low in the external cohorts, PPV was generally low, whereas NPV remained consistently high. In MIMIC-III, ANN achieved the highest AUC of 0.830, whereas RF, SVM, and XGBoost showed comparable AUCs of 0.827, 0.821, and 0.820, respectively. Although XGBoost did not achieve the highest AUC in this cohort, it had the highest sensitivity among these high-performing models (0.877) and maintained favorable accuracy, F1 score, Cohen’s kappa, and Youden’s index, indicating stable identification of patients at high risk of postoperative sepsis. Its Brier score was 0.213, indicating acceptable overall prediction error, while the calibration curve showed some deviation between predicted and observed risks(**Supplementary Figure 7**).

In the eICU cohort, XGBoost achieved the highest AUC of 0.837 (95% CI, 0.788–0.886) and showed a favorable balance among sensitivity, specificity, and F1 score. It also had the lowest Brier score of 0.196, indicating good agreement between predicted and observed risks (**Supplementary Figure 8**). In the JPHITCWM cohort, XGBoost achieved the highest AUC of 0.796 (95% CI, 0.724–0.862). The corresponding Brier score was 0.440, and the calibration curve showed greater deviation from the ideal reference line than those in the other validation cohorts (**Supplementary Figure 9**), indicating cohort-specific variation in probability calibration. DeLong tests were used to compare XGBoost with other high-performing models in the test set and MIMIC-III cohort. In the test set, the AUC difference between XGBoost and RF was not statistically significant (0.794 vs. 0.802; z = −1.34, P = 0.181). In MIMIC-III, XGBoost showed no significant AUC differences compared with RF, SVM, or ANN (P = 0.343, 0.942, and 0.441, respectively), indicating discrimination comparable to that of other high-performing models. Considering discrimination, calibration, classification performance, and decision-curve net benefit across cohorts rather than AUC alone, XGBoost was selected as the primary model for subsequent interpretation and clinical risk stratification. XGBoost maintained broadly acceptable discrimination across datasets, although its threshold-dependent classification performance and probability calibration varied across cohorts. Calibration curves showed cohort-dependent patterns, particularly in the JPHITCWM cohort. Decision curve analysis assessed the clinical utility of each model across different threshold probabilities. Overall, most models showed net benefit over the treat-all and treat-none strategies within certain threshold ranges, suggesting potential clinical utility for postoperative sepsis risk stratification. XGBoost showed net benefit across multiple validation cohorts; however, the magnitude and applicable threshold ranges varied, with relatively limited incremental net benefit in the JPHITCWM cohort. Therefore, DCA results should be interpreted cautiously according to specific threshold ranges and clinical scenarios (**Supplementary Figures 10**-**14**).Confusion matrix analysis further characterized the threshold-dependent classification performance of XGBoost across the validation cohorts. Classification metrics at this fixed threshold varied across cohorts. It achieved balanced classification in the test cohort and reasonable recognition of postoperative sepsis cases in the MIMIC-III and eICU external validation cohorts (**Figure 5**, **Supplementary Figure 16**).

**Figure 5:**
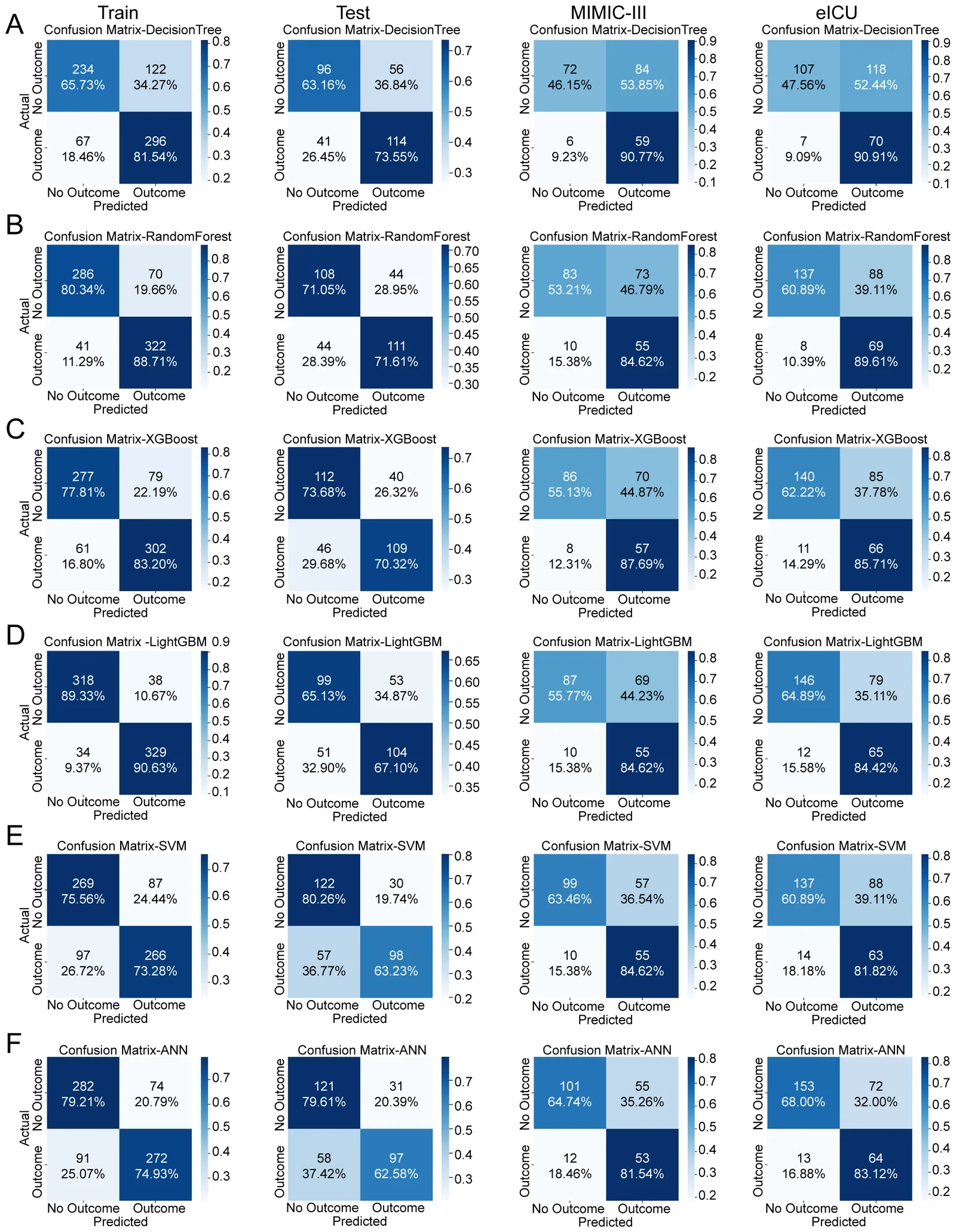
Confusion matrices of six machine learning models across four datasets. From left to right, the columns correspond to the MIMIC-IV training set, MIMIC-IV internal test set, MIMIC-III external validation cohort, and eICU external validation cohort. From top to bottom, the rows correspond to **(A)** decision tree, **(B)** random forest, **(C)** XGBoost, **(D)** LightGBM, **(E)** support vector machine, and **(F)** artificial neural network. Each confusion matrix presents the distribution of true negatives, false positives, false negatives, and true positives, together with the corresponding row percentages, enabling direct comparison of classification accuracy and error patterns across models and datasets.

Although some models showed comparable performance in the test set or individual external validation cohorts, XGBoost demonstrated comparatively balanced performance across cohorts. It achieved the highest AUCs in both the eICU and JPHITCWM cohorts, while DeLong tests showed no significant differences from other high-performing models in the test set and MIMIC-III cohort. Its Brier scores and calibration curves showed cohort-dependent probability performance, while decision curve analysis indicated potential net benefit within certain threshold ranges. Considering discrimination, calibration, classification performance, and clinical net benefit, XGBoost was ultimately selected as the primary prediction model.

### Model interpretability

To improve model interpretability, SHAP and LIME analyses were performed for the XGBoost model to quantify predictor contributions from both global and individual perspectives. Global feature importance based on mean absolute SHAP values showed that SOFA score contributed most to model output, followed by glucose, vasopressor use, pneumonia, WBC, sedative use, mannitol use, GCS score, mechanical ventilation, SpO_2_, chloride, RBC, and aspirin use. This structured ranking quantified the relative contributions of routinely available indicators of organ dysfunction, metabolic abnormalities, circulatory support, infection status, and inflammation to model predictions. The SHAP-based interpretability results are presented in **Figure 6**.

**Figure 6:**
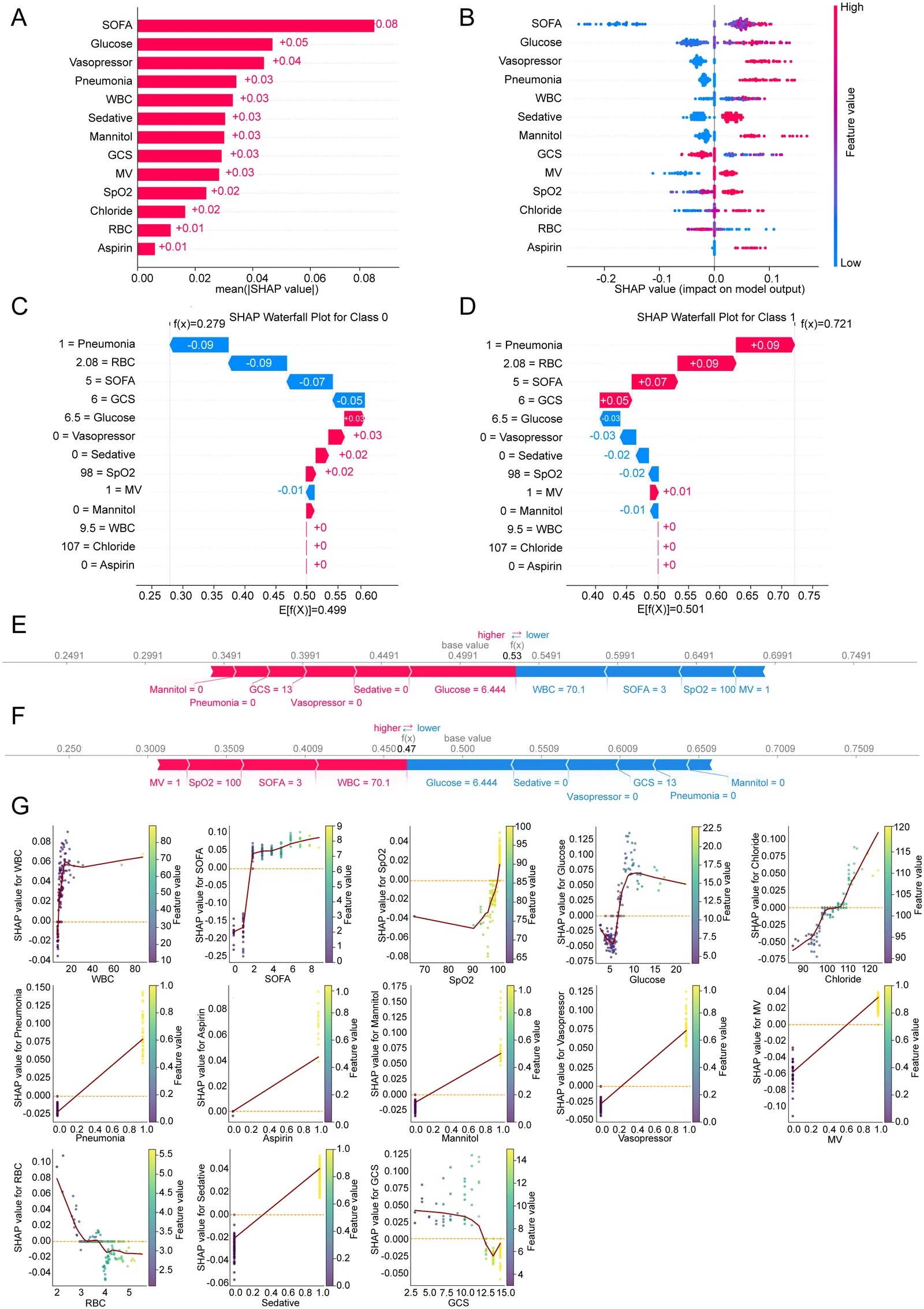
SHAP-based interpretability visualizations for the XGBoost model. **(A)** SHAP bar plot showing global feature importance based on mean absolute SHAP values. **(B)** SHAP beeswarm plot showing the direction and magnitude of predictor effects. **(C, D)** SHAP waterfall plots illustrating predictor contributions to representative class 0 and class 1 predictions, respectively. **(E, F)** SHAP force plots illustrating individual-level predictor contributions to representative predictions. **(G)** SHAP dependence plots showing potential nonlinear effects of the thirteen final predictors. Together, these analyses provide global and individualized explanations of the model.

The SHAP bees warm plot illustrated the direction of each variable’s effect on model output. Higher SOFA score, glucose, WBC, and chloride, as well as vasopressor use, pneumonia, sedative use, mannitol use, and mechanical ventilation, were generally associated with positive SHAP values, indicating higher predicted postoperative sepsis risk. In contrast, higher GCS score and RBC tended to be associated with lower predicted risk. SHAP dependence plots showed nonlinear associations between continuous variables, including SOFA score, glucose, WBC, chloride, SpO_2_, RBC, and GCS score, and model output, suggesting that XGBoost captured complex variable–risk relationships.

At the individual level, SHAP waterfall and force plots decomposed the direction and magnitude of each variable’s contribution to single-patient predictions. In the high-risk example, pneumonia, lower RBC, SOFA score, GCS score, and glucose jointly increased predicted risk, whereas in the low-risk example, these variables collectively decreased predicted risk. These individual-level explanations improved the transparency of XGBoost predictions and supported individualized postoperative sepsis risk assessment.

LIME analysis was performed as a complementary interpretability method to SHAP (**Supplementary Figure 15**). In a representative high-risk patient, XGBoost predicted a 91% probability of postoperative sepsis. Feature weights showed that SOFA score range, vasopressor use, pneumonia, mechanical ventilation, sedative use, aspirin use, higher SpO_2_, and higher chloride contributed to the high-risk classification, whereas no mannitol use and lower glucose contributed in the opposite direction.

### Sensitivity analysis

After excluding baseline SOFA, all six machine learning models were retrained using the remaining 12 predictors. The magnitude and direction of changes in AUC varied across algorithms (**Supplementary Table 7**). For XGBoost, the AUC was 0.757 (95% CI, 0.699–0.805) in the internal test set and 0.811 (95% CI, 0.749–0.865), 0.823 (95% CI, 0.777–0.871), and 0.772 (95% CI, 0.699–0.844) in the MIMIC-III, eICU, and JPHITCWM cohorts, respectively. Compared with the original XGBoost model, the AUC difference was statistically significant in the internal test set (ΔAUC = −0.037; P = 0.001), but not in MIMIC-III (ΔAUC = −0.009; P = 0.458), eICU (ΔAUC = −0.014; P = 0.226), or JPHITCWM (ΔAUC = −0.024; P = 0.081). Complete discrimination, calibration, and classification results for the SOFA-free models are provided in **Supplementary Table 8**. The ROC curves of the six SOFA-free models in the MIMIC-IV training set, MIMIC-IV internal test set, MIMIC-III, eICU, and JPHITCWM cohorts are presented in **Supplementary Figures 19**-**23**. SHAP analysis of the SOFA-free XGBoost model identified GCS as the leading contributor to model predictions, followed by glucose, pneumonia, vasopressor administration, and WBC (**Supplementary Figure 24**). Glucose, pneumonia, vasopressor administration, and WBC remained among the leading contributors in both the original and SOFA-free models, whereas the relative contribution of GCS increased after SOFA exclusion. These findings suggest that the external discrimination of XGBoost was not solely dependent on baseline SOFA. However, the increased contribution of GCS indicates that some severity-related predictive information may have been redistributed among the remaining variables after SOFA exclusion.

### Implementation of the web calculator

The final XGBoost model was deployed as an interactive web-based prediction tool (**Figure 7**). The interface requires input of routinely available early ICU variables, including SOFA score, glucose, vasopressor use, pneumonia, WBC, sedative use, SpO_2_, chloride, GCS score, mannitol use, aspirin use, mechanical ventilation, and RBC. After submission, the tool provides an individualized prediction of postoperative sepsis risk, accompanied by graphical explanations based on the model output.

**Figure 7:**
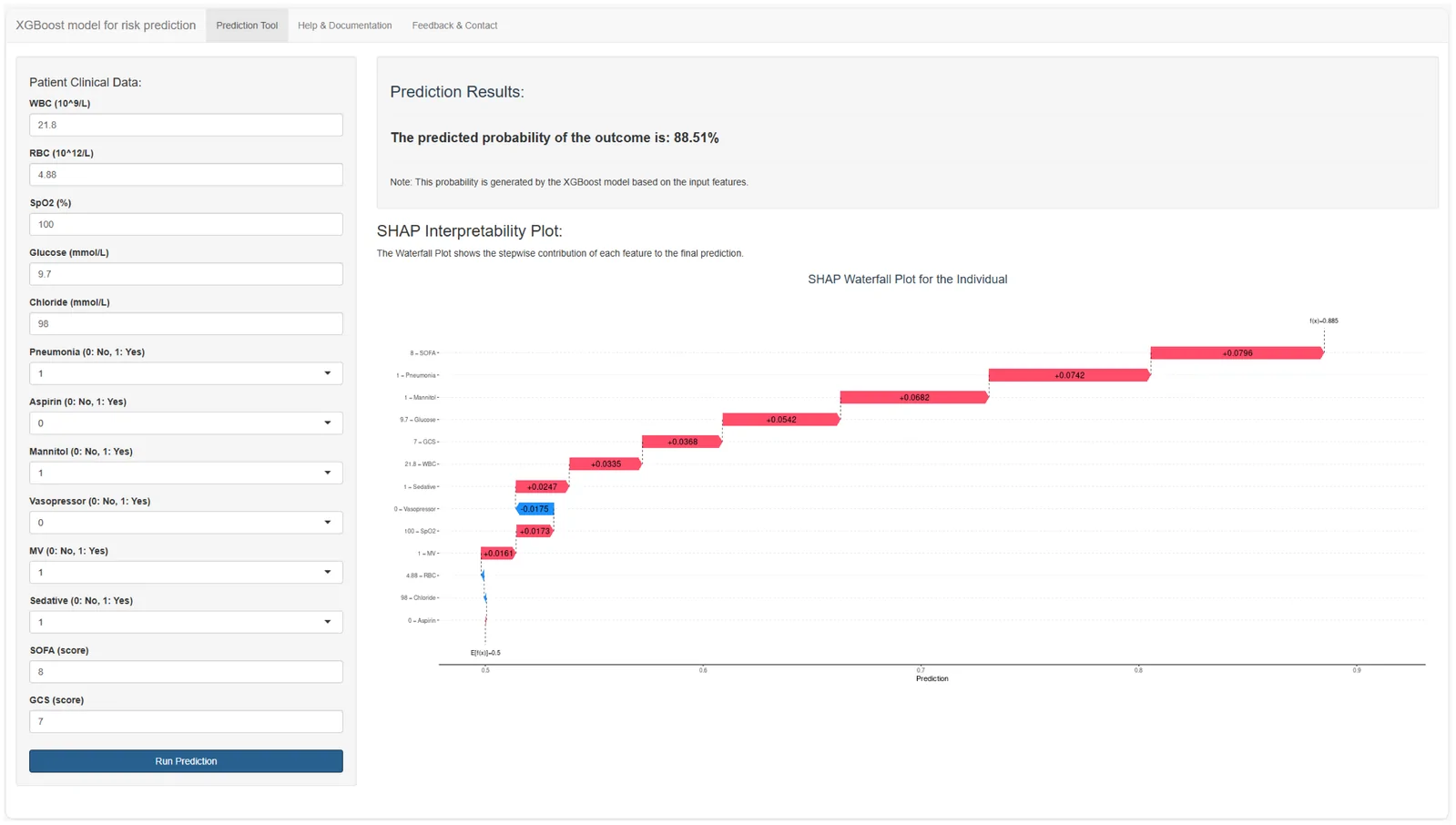
A web-based calculator for predicting postoperative sepsis risk in ICU patients after intracranial hematoma evacuation.

A web-based application (https://predi2.shinyapps.io/shiny_web/) was developed using the R package “shiny” to make the model accessible online. The interactive interface provides convenient access to individualized model estimates and graphical explanations. However, because the calculator directly implements the retrospectively developed model, it should be considered a research-use prototype rather than a validated clinical decision support system. Its outputs should be interpreted alongside clinical assessment, and prospective implementation and clinical impact evaluation are required before routine clinical use.

## Discussion

Based on multicenter real-world data, this study developed and validated an interpretable machine learning model to predict sepsis risk in ICU patients after intracranial hematoma evacuation. The study integrated three large critical care databases—MIMIC-IV, MIMIC-III, and eICU—and an independent clinical validation cohort from Jiangsu Provincial Hospital of Integrated Traditional Chinese and Western Medicine, covering patients across different time periods, healthcare systems, and real-world clinical settings. The outcome was defined according to the operationalized Sepsis-3 criteria, namely an acute increase in SOFA score of ≥2 points in the context of infection (Singer et al., 2016). Among the six algorithms, XGBoost showed broadly consistent discrimination across the evaluated cohorts and achieved the highest AUCs in the eICU and JPHITCWM cohorts. Calibration curves and Brier scores demonstrated cohort-dependent probability performance, while decision curve analysis showed potential net benefit within certain threshold ranges across multiple cohorts. Considering these complementary dimensions rather than AUC alone, XGBoost was selected as the primary model. SHAP and LIME analyses were applied to enhance model transparency, and an interactive web-based prediction tool was developed to facilitate individualized postoperative sepsis risk assessment (Goecks et al., 2020).

In this study, XGBoost maintained acceptable discrimination in the test set and external validation cohorts. Previous sepsis prediction studies have mainly focused on general ICU or mixed medical-surgical populations. For example, models for early septic shock prediction have been developed using MIMIC-IV and eICU-CRD, but the target population remained a broad critical care population (Zhou et al., 2026). In postoperative or perioperative settings, existing studies have also tended to include broadly defined surgical populations. One study developed a prognostic model for critically ill major surgical patients and included perioperative variables such as sepsis complications, but its outcomes focused mainly on mortality or prolonged ICU stay rather than postoperative sepsis as a specific complication (Zhang et al., 2025). In neurocritical care, machine learning studies have more often focused on isolated intracerebral hemorrhage or traumatic brain injury. Liu et al. developed a sepsis prediction model for ICU patients with intracerebral hemorrhage using MIMIC-IV and externally validated it in eICU (Liu et al., 2025). Another study by Liu et al. focused on traumatic brain injury and developed an interpretable machine learning model using MIMIC-IV with validation in eICU-CRD (Liu et al., 2024). These studies provide an important foundation, but they mainly focused on single disease subgroups rather than the clinically distinct, high-exposure population after intracranial hematoma evacuation. Some previous models were also limited by small sample sizes, heterogeneous populations, or insufficient external validation.

Importantly, the novelty of this study lies in its focus on a procedure-defined postoperative neurocritical care population rather than a general ICU or single-diagnosis cohort. Despite heterogeneous hematoma etiologies, these patients share a clinical context in which acute neurological injury, impaired consciousness, and postoperative organ-support requirements may jointly influence the risk of infection and subsequent sepsis. Focusing on this shared postoperative context may improve the clinical relevance of risk stratification for postoperative monitoring Compared with models developed from a single center or database, this study used a more comprehensive cross-database validation framework. In addition to model development and testing using a 70%/30% split of MIMIC-IV, external validation was performed in MIMIC-III, eICU, and the independent JPHITCWM cohort. MIMIC-III provided temporal validation, eICU provided geographical validation across institutions and healthcare systems, and the institutional cohort provided an additional evaluation in a real-world clinical setting. This design enabled a broader assessment of cross-cohort performance and potential model transportability (Austin et al., 2016; de Hond et al., 2023). Sepsis incidence varied across cohorts, ranging from 25.5% in eICU to 50.5% in MIMIC-IV, which may reflect differences in patient case mix, disease severity, healthcare settings, infection surveillance, and clinical management. Such variations may affect calibration and threshold-dependent measures, particularly PPV and NPV, even when AUC remains relatively stable. These variations should therefore be considered when interpreting model performance across cohorts and applying the model in different clinical settings.

The 13 predictors selected from 38 candidate variables covered several key clinical domains: organ dysfunction and physiological disturbance (SOFA, SpO_2_, and chloride), circulatory support and treatment intensity (vasopressor use, mechanical ventilation, and sedative use), infection and inflammation (pneumonia and WBC), and neurological injury severity and brain-protective therapy (GCS and mannitol), together with aspirin use and RBC. This variable profile suggests that postoperative sepsis risk is driven by multiple interacting clinical domains rather than a single inflammatory marker. This pattern is consistent with current understanding of infection susceptibility in neurocritical care patients. After intracranial hematoma evacuation, infection and secondary sepsis may arise from brain injury–related immune dysregulation and healthcare-related exposures. Acute brain injury can disrupt neuroimmune homeostasis through autonomic and neuroendocrine pathways, leading to early systemic inflammation followed by brain injury–associated immunosuppression (Simon et al., 2017; Faura et al., 2021). Impaired consciousness may weaken airway protection, while pneumonia, mechanical ventilation, sedation, and other invasive procedures may increase the risk of hospital-acquired infection and secondary sepsis (Singer et al., 2016; Howroyd et al., 2024). SOFA score and vasopressor requirement reflect circulatory instability and high-risk physiological states (Monnet et al., 2025). Hyperglycemia may indicate stress-related metabolic disturbance and impaired immune function (Turina et al., 2005; Kulikov et al., 2022). Electrolyte disturbances, anemia, or transfusion-related immune modulation may also contribute to infection susceptibility and sepsis progression (Athar et al., 2012; Filis et al., 2018). Previous studies have also linked lower GCS scores with greater clinical complexity and higher sepsis risk in neurocritical care patients (Zhou et al., 2022). Feature selection was conducted in the MIMIC-IV training cohort, and the resulting 13-predictor set was applied unchanged to all validation cohorts to preserve independent model evaluation. The preservation of predictive performance across cohorts provides some reassurance regarding the applicability of the fixed feature set. However, differences in case mix and predictor distributions may influence the relative contributions of individual features; therefore, cross-cohort feature stability should be interpreted cautiously and further examined in future multicenter studies.

Because ensemble models are often difficult to interpret, SHAP and LIME were used to explain XGBoost at both global and individual levels (Wongvibulsin et al., 2020; Hettikankanamage et al., 2025). In neurocritical care, postoperative sepsis risk arises from complex interactions among organ dysfunction, treatment exposure, infection burden, and metabolic disturbance, which may not be fully captured by traditional linear models or single scoring systems (Naik et al., 2024; Xu et al., 2025). SHAP analysis identified SOFA score, glucose, vasopressor use, pneumonia, WBC, and sedative exposure as the main contributors to XGBoost predictions. These clinically plausible findings should be interpreted as a structured, model-specific quantification of established risk-related clinical information across organ dysfunction, circulatory support, metabolic abnormalities, infection status, and inflammation, rather than as the identification of novel etiological risk factors (Nseir et al., 2010; Singer et al., 2016; Modi and Kovacs, 2020; Evans et al., 2021). SHAP also showed nonlinear associations between model output and variables such as SOFA score, glucose, and WBC, whereas higher GCS and RBC were generally associated with lower predicted risk. LIME explanations were broadly consistent with SHAP findings, further supporting the interpretability and clinical coherence of the model. Tree-based ensemble models are generally less affected by conventional multicollinearity than regression models; however, correlated predictors may be used interchangeably as split variables and redistribute SHAP attribution. In this study, VIF assessment showed no substantial linear multicollinearity, while Spearman analysis identified no strong pairwise correlations among the 13 final predictors. Nevertheless, the increased SHAP contribution of GCS after SOFA exclusion suggests that some severity-related information may have been redistributed among the remaining variables. Therefore, SHAP values should be interpreted as model-specific predictive contributions rather than independent or causal effects.

The substantially higher baseline SOFA score in the JPHITCWM cohort indicated an important severity-related case-mix shift. To examine whether the observed predictive performance primarily reflected general disease severity, we retrained all six models after excluding baseline SOFA. Although the XGBoost AUC decreased in the internal test set, only modest and statistically nonsignificant changes were observed in the three external validation cohorts, including JPHITCWM. These findings suggest that the external discrimination of XGBoost was not solely attributable to the aggregate SOFA score, while the potential contribution of residual severity-related information should still be considered. Nevertheless, several remaining predictors, including GCS, vasopressor use, mechanical ventilation, SpO_2_, and sedative exposure, may also reflect disease severity or treatment intensity. Therefore, the influence of general clinical deterioration cannot be completely excluded. Accordingly, XGBoost may complement established clinical severity scores by integrating multidimensional clinical information for postoperative sepsis risk stratification, while its incremental value beyond these scores warrants further evaluation. Beyond the overlap between baseline SOFA and the predictor set, the Sepsis-3 definition itself requires cautious interpretation in postoperative neurosurgical patients. Reduced GCS may reflect primary brain injury or sedation, while respiratory and cardiovascular SOFA abnormalities may partly result from mechanical ventilation, perioperative hemodynamic support, or other postoperative factors. Acute brain injury and surgical stress may also produce noninfectious systemic inflammation resembling infection-related deterioration. Therefore, potential misclassification of infection-related organ dysfunction cannot be completely excluded, and model predictions should be interpreted alongside clinical assessment.

SHAP and LIME provided both global feature rankings and individual-level explanations. This helped translate “black-box” predictions into traceable and clinically interpretable information. For clinicians, this framework may help clarify whether a high-risk prediction is mainly driven by organ dysfunction, infection exposure, metabolic abnormalities, or increased treatment intensity (Pinsky et al., 2024; Athukorala and Ilmini, 2026). Because all model variables are routinely available early after ICU admission, the risk estimate could be generated once the initial clinical data become available and displayed through an electronic health record or bedside dashboard. A high-risk result could prompt enhanced clinical monitoring and earlier structured assessment for potential respiratory, bloodstream, urinary, or surgical-site infection, with microbiological testing or imaging performed when clinically indicated. The web calculator reproduces the output of the fixed model and therefore inherits its limitations, including cross-cohort variation in calibration and decision thresholds. It should be regarded as a research-use prototype rather than a validated clinical decision support system. The model should complement clinical judgment rather than automatically trigger antimicrobial treatment (Efthimiou et al., 2024; Collins et al., 2024).Prospective implementation studies are needed to determine appropriate alert thresholds and evaluate its effects on clinical workflow and patient outcomes.

This study has several strengths, including its multidata base design, comparison of multiple algorithms, temporal and geographical external validation, and emphasis on interpretability. However, several limitations should be noted. First, the retrospective design and eligibility criteria may have introduced survivor and selection bias. Requiring an ICU stay of more than 24 hours may have excluded patients who died early or were discharged rapidly, while restricting the analysis to first ICU admissions and patients with sufficiently complete clinical data may have further limited cohort representativeness. Consequently, the study population may not fully represent all patients undergoing intracranial hematoma evacuation, particularly those with very early mortality or short ICU stays. Information bias and unmeasured confounding also cannot be excluded; therefore, the findings reflect predictive associations rather than causal relationships (Moons et al., 2025). Second, to support early prediction, the model used first recorded values after ICU admission and did not incorporate dynamic information, such as serial SOFA scores, WBC or glucose trends, or ventilation trajectories. Longitudinal modeling may improve early warning performance in future studies (Islam et al., 2023). Third, SOFA was both a predictor and part of the Sepsis-3 framework, which may introduce definitional overlap. Furthermore, in postoperative neurosurgical patients, changes in SOFA may partly reflect primary neurological injury, sedation, mechanical ventilation, vasopressor support, or other perioperative factors rather than infection-related organ dysfunction alone, potentially contributing to outcome misclassification. Although the SOFA-free models generally retained moderate-to-good discrimination, their performance changes varied across algorithms and cohorts. Moreover, several remaining physiological and treatment-related predictors may still reflect general disease severity. Therefore, residual severity-related confounding cannot be completely excluded (Singer et al., 2016). Fourth, although external validation was performed in MIMIC-III, eICU, and the independent JPHITCWM cohort, model development and validation still relied mainly on US ICU databases, and the domestic validation cohort was from a single center. Further multicenter recalibration and prospective validation are needed before broader clinical implementation (Riley et al., 2016; Collins et al., 2024).Fifth, Detailed neurosurgical and perioperative information, such as hematoma location and volume, surgical approach, operative duration, intraoperative blood loss, intracranial pressure monitoring, postoperative rebleeding, and residual hematoma burden, was unavailable in the public ICU databases. These factors may affect postoperative neurological severity, treatment intensity, and infection susceptibility, and their absence may have limited model adjustment and interpretability. Future studies integrating operative records and neuroimaging features may further improve sepsis risk prediction in this population. In addition, administrative codes were used to define the study population, which may introduce misclassification. Although diagnosis codes for intracranial hemorrhage or hematoma were combined with procedure codes related to hematoma evacuation or drainage to improve specificity, some codes may represent catheter-related neurosurgical procedures associated with hematoma management rather than procedures exclusively performed for hematoma evacuation. Residual heterogeneity in surgical management therefore cannot be fully excluded. Although VIF and pairwise correlation analyses did not indicate severe multicollinearity, these methods cannot exclude nonlinear or conditional dependencies among predictors. In addition, physiological and treatment-related variables containing overlapping clinical information may redistribute SHAP attribution. Therefore, the relative importance and ranking of individual predictors should be interpreted cautiously and should not be regarded as evidence of independent biological or causal effects. Finally, although the model underwent retrospective external validation, the web calculator remains a research-use implementation of the fixed model and therefore inherits its limitations. Neither the model nor the calculator has been prospectively evaluated for clinical utility or effects on patient outcomes. Decision thresholds may vary across institutions because of differences in sepsis prevalence, model calibration, and the relative consequences of missed cases and false alerts. Integration into clinical decision-making also requires evaluation of workflow burden, alert fatigue, clinician responses, and potential overreliance on model outputs; therefore, prospective implementation studies are required before routine clinical use (Islam et al., 2023). Future studies should incorporate broader multicenter data, dynamic clinical information, and prospective implementation designs to refine model updating, external evaluation, and clinical translation.

## Conclusions

This study developed and externally validated an XGBoost-based machine learning model to predict sepsis risk in ICU patients after intracranial hematoma evacuation using multicenter real-world data, with further validation in an independent clinical cohort. Among the six algorithms, XGBoost showed a comparatively balanced overall performance profile across the evaluated cohorts. SHAP and LIME analyses identified early available variables, including SOFA score, glucose, vasopressor use, pneumonia, WBC, and sedative exposure, as leading contributors to model predictions. This model may serve as a complementary tool for early identification and individualized risk stratification of sepsis in ICU patients after intracranial hematoma evacuation, although further validation in larger multicenter prospective studies is needed.

## Data Availability

The data analyzed in this study are subject to the following licenses and restrictions. MIMIC-IV, MIMIC-III, and the eICU Collaborative Research Database are available through PhysioNet after completion of the required training and data-use agreements. Data from the independent clinical cohort are not publicly available because of institutional privacy and ethical restrictions but may be made available by the authors upon reasonable request and approval by the relevant Ethics Committee. Requests should be directed to Yong Zhang (zhangyong@jsatcm.com). The web-based prediction tool developed in this study is publicly accessible at https://predi2.shinyapps.io/shiny_web/.
