# Supplementary material for: Interpretable machine learning for postoperative sepsis prediction in ICU patients following intracranial hematoma evacuation
Source: Front Cell Infect Microbiol. 2026 Sep 22;16:1895258. doi: 10.3389/fcimb.2026.1895258 (PMC13638375; doi:10.3389/fcimb.2026.1895258)
Supplement: Supplementary file 1 [file DataSheet1.docx]

**Table S1: Collinearity Analysis of Candidate and Selected Variables.**

| **Variable** | **VIF (All candidate variables)** | **VIF (Selected features)** | **Inclusion in final model** |
| --- | --- | --- | --- |
| Sex | 1.43353492864039 |  |  |
| **MV** | 1.20178049092943 | 1.09680925829564 | Yes |
| **Vasopressor** | 1.38697274733741 | 1.23477146077964 | Yes |
| CRRT | 1.13038797266345 |  |  |
| **Mannitol** | 1.2079654482086 | 1.09166843879443 | Yes |
| **Aspirin** | 1.06781856327379 | 1.00978380069151 | Yes |
| Heparin | 1.1211065061162 |  |  |
| **Sedative** | 1.27829943382484 | 1.19978914463172 | Yes |
| Analgesic | 1.1276080290673 |  |  |
| Hypertension | 1.36159332875429 |  |  |
| HF | 1.46388128385615 |  |  |
| Diabetes | 1.26115757655837 |  |  |
| CKD | 1.58350700858917 |  |  |
| **Pneumonia** | 1.20140918739931 | 1.14037636387738 | Yes |
| Coagulopathy | 1.14927947105548 |  |  |
| Age | 1.67081534683934 |  |  |
| Weight | 1.38860760027937 |  |  |
| **SOFA** | 1.9516375744183 | 1.53960637655101 | Yes |
| **GCS** | 1.57324076018159 | 1.40517136913769 | Yes |
| HR | 1.20906075368759 |  |  |
| SBP | 3.74421540373884 |  |  |
| DBP | 14.3107942281948 |  |  |
| MAP | 20.8247394807589 |  |  |
| **SpO2** | 1.17335626670758 | 1.06450003192937 | Yes |
| **Glucose** | 1.38388599148305 | 1.09306488978176 | Yes |
| **Chloride** | 1.33497158507891 | 1.07575892613632 | Yes |
| Potassium | 1.34326283138927 |  |  |
| BUN | 1.61748603632935 |  |  |
| Hematocrit | 607.447438847898 |  |  |
| Hemoglobin | 611.903921144045 |  |  |
| RDW | 1.66279228227238 |  |  |
| Platelet | 1.36350593884254 |  |  |
| **WBC** | 1.31993067074518 | 1.0986018407267 | Yes |
| **RBC** | 140.028762103122 | 1.11178835751238 | Yes |
| Bicarbonate | 1.46558963371727 |  |  |
| Calcium | 1.55278796660682 |  |  |
| MCHC | 40.9177041650343 |  |  |
| MCV | 23.8445711537575 |  |  |

**Table S2. Baseline Characteristics of ICU Patients Undergoing Surgical Evacuation of Intracranial Hematoma in the MIMIC-IV Database According to Postoperative Sepsis Status**

| **Characteristics** | **level** | **Overall**  **(n=1026)** | **Nonsepsis**  **(n=508)** | **Sepsis(n=518)** | ***p*** |
| --- | --- | --- | --- | --- | --- |
| Sex | Female | 346 (33.72) | 170 (33.46) | 176 (33.98) | 0.914 |
|  | Male | 680 (66.28) | 338 (66.54) | 342 (66.02) |  |
| MV | No | 274 (26.71) | 195 (38.39) | 79 (15.25) | <0.001 |
|  | Yes | 752 (73.29) | 313 (61.61) | 439 (84.75) |  |
| Vasopressor | No | 793 (77.29) | 462 (90.94) | 331 (63.90) | <0.001 |
|  | Yes | 233 (22.71) | 46 ( 9.06) | 187 (36.10) |  |
| CRRT | No | 1014 (98.83) | 507 (99.80) | 507 (97.88) | 0.01 |
|  | Yes | 12 ( 1.17) | 1 ( 0.20) | 11 ( 2.12) |  |
| Mannitol | No | 881 (85.87) | 475 (93.50) | 406 (78.38) | <0.001 |
|  | Yes | 145 (14.13) | 33 ( 6.50) | 112 (21.62) |  |
| Aspirin | No | 965 (94.05) | 493 (97.05) | 472 (91.12) | <0.001 |
|  | Yes | 61 ( 5.95) | 15 ( 2.95) | 46 ( 8.88) |  |
| Heparin | No | 106 (10.33) | 70 (13.78) | 36 ( 6.95) | <0.001 |
|  | Yes | 920 (89.67) | 438 (86.22) | 482 (93.05) |  |
| Sedative | No | 534 (52.05) | 338 (66.54) | 196 (37.84) | <0.001 |
|  | Yes | 492 (47.95) | 170 (33.46) | 322 (62.16) |  |
| Analgesic | No | 42 ( 4.09) | 31 ( 6.10) | 11 ( 2.12) | 0.002 |
|  | Yes | 984 (95.91) | 477 (93.90) | 507 (97.88) |  |
| Hypertension | No | 461 (44.93) | 218 (42.91) | 243 (46.91) | 0.221 |
|  | Yes | 565 (55.07) | 290 (57.09) | 275 (53.09) |  |
| HF | No | 927 (90.35) | 468 (92.13) | 459 (88.61) | 0.072 |
|  | Yes | 99 ( 9.65) | 40 ( 7.87) | 59 (11.39) |  |
| Diabetes | No | 783 (76.32) | 397 (78.15) | 386 (74.52) | 0.195 |
|  | Yes | 243 (23.68) | 111 (21.85) | 132 (25.48) |  |
| CKD | No | 931 (90.74) | 464 (91.34) | 467 (90.15) | 0.585 |
|  | Yes | 95 ( 9.26) | 44 ( 8.66) | 51 ( 9.85) |  |
| Pneumonia | No | 849 (82.75) | 475 (93.50) | 374 (72.20) | <0.001 |
|  | Yes | 177 (17.25) | 33 ( 6.50) | 144 (27.80) |  |
| Coagulopathy | No | 821 (80.02) | 420 (82.68) | 401 (77.41) | 0.042 |
|  | Yes | 205 (19.98) | 88 (17.32) | 117 (22.59) |  |
| Age(years)(median [IQR]) |  | 70.00 [59.00, 80.00] | 72.00 [63.00, 81.00] | 68.00 [56.00, 79.00] | 0.003 |
| Weight(kg) (median [IQR]) |  | 75.32 [63.90, 87.10] | 76.10 [63.60, 87.03] | 74.80 [64.00, 87.10] | 0.978 |
| SOFA (median [IQR]) |  | 3.00 [2.00, 4.00] | 2.00 [1.00, 3.00] | 3.00 [2.00, 5.00] | <0.001 |
| GCS (median [IQR]) |  | 14.00 [12.00, 15.00] | 14.00 [13.00, 15.00] | 14.00 [10.00, 15.00] | <0.001 |
| HR(bpm)(median [IQR]) |  | 77.00 [67.00, 89.00] | 76.00 [66.00, 88.00] | 79.00 [69.00, 90.00] | 0.003 |
| SBP(mmHg)(median [IQR]) |  | 129.00 [115.00, 144.00] | 130.00 [117.00, 144.00] | 129.00 [112.00, 143.00] | 0.322 |
| DBP(mmHg)(median [IQR]) |  | 70.00 [61.00, 82.00] | 71.00 [62.00, 83.00] | 69.00 [59.00, 80.00] | 0.026 |
| MAP(mmHg)(median [IQR]) |  | 85.00 [76.00, 97.00] | 87.00 [78.75, 97.00] | 84.00 [74.00, 96.00] | 0.002 |
| SpO2(%)(median [IQR]) |  | 99.00 [97.00, 100.00] | 98.00 [96.00, 100.00] | 100.00 [97.00, 100.00] | <0.001 |
| Glucose(mmol/L)(median [IQR]) |  | 6.94 [5.78, 8.72] | 6.47 [5.54, 7.83] | 7.44 [6.17, 9.50] | <0.001 |
| Chloride(mmol/L)(median [IQR]) |  | 104.00 [101.00, 107.00] | 104.00 [101.00, 107.00] | 105.00 [102.00, 108.00] | 0.005 |
| Potassium(mmol/L)(median [IQR]) |  | 3.90 [3.60, 4.30] | 4.00 [3.70, 4.30] | 3.90 [3.60, 4.30] | 0.083 |
| BUN(mg/dL)(median [IQR]) |  | 14.00 [11.00, 19.00] | 14.00 [10.00, 18.00] | 15.00 [11.00, 19.00] | 0.021 |
| Hematocrit(%)(median [IQR]) |  | 34.48 (5.79) | 35.53 (5.31) | 33.44 (6.05) | <0.001 |
| Hemoglobin(g/dL)(mean (SD)) |  | 11.46 (2.03) | 11.81 (1.88) | 11.12 (2.11) | <0.001 |
| RDW(%)(median [IQR]) |  | 13.60 [12.90, 14.80] | 13.40 [12.80, 14.30] | 14.00 [13.20, 15.20] | <0.001 |
| Platelet(×10⁹/L)(median [IQR]) |  | 202.00 [156.25, 255.00] | 207.50 [169.00, 256.50] | 191.00 [144.25, 252.75] | 0.002 |
| WBC(×10⁹/L)(median [IQR]) |  | 9.20 [6.90, 12.10] | 8.70 [6.80, 11.20] | 9.85 [7.12, 13.00] | <0.001 |
| RBC(×10¹²/L)(mean (SD)) |  | 3.80 (0.70) | 3.91 (0.63) | 3.69 (0.74) | <0.001 |
| Bicarbonate(mmol/L)(median [IQR]) |  | 23.00 [21.00, 25.00] | 23.00 [22.00, 25.00] | 23.00 [21.00, 25.00] | 0.032 |
| Calcium(mg/dL)(median [IQR]) |  | 8.65 [8.20, 9.10] | 8.70 [8.30, 9.10] | 8.60 [8.03, 9.00] | 0.002 |
| MCHC(g/dL)(median [IQR]) |  | 33.20 [32.40, 34.20] | 33.20 [32.40, 34.10] | 33.20 [32.20, 34.20] | 0.906 |
| MCV(fL)(median [IQR]) |  | 92.00 [87.00, 95.00] | 92.00 [88.00, 95.00] | 92.00 [87.00, 96.00] | 0.861 |

**Table S3. Baseline Characteristics of ICU Patients Undergoing Surgical Evacuation of Intracranial Hematoma in the MIMIC-III Database According to Postoperative Sepsis Status**

| **Characteristics** | **level** | **Overall**  **(n=221)** | **Nonsepsis**  **(n=156)** | **Sepsis (n=65)** | ***p*** |
| --- | --- | --- | --- | --- | --- |
| Sex | Female | 75 ( 33.94) | 51 ( 32.69) | 24 ( 36.92) | 0.653 |
|  | Male | 146 ( 66.06) | 105 ( 67.31) | 41 ( 63.08) |  |
| MV | No | 106 ( 47.96) | 98 ( 62.82) | 8 ( 12.31) | <0.001 |
|  | Yes | 115 ( 52.04) | 58 ( 37.18) | 57 ( 87.69) |  |
| Vasopressor | No | 169 ( 76.47) | 136 ( 87.18) | 33 ( 50.77) | <0.001 |
|  | Yes | 52 ( 23.53) | 20 ( 12.82) | 32 ( 49.23) |  |
| CRRT | No | 221 (100.00) | 156 (100.00) | 65 (100.00) | NA |
| Mannitol | No | 169 ( 76.47) | 132 ( 84.62) | 37 ( 56.92) | <0.001 |
|  | Yes | 52 ( 23.53) | 24 ( 15.38) | 28 ( 43.08) |  |
| Aspirin | No | 215 ( 97.29) | 152 ( 97.44) | 63 ( 96.92) | 1 |
|  | Yes | 6 ( 2.71) | 4 ( 2.56) | 2 ( 3.08) |  |
| Heparin | No | 24 ( 10.86) | 23 ( 14.74) | 1 ( 1.54) | 0.008 |
|  | Yes | 197 ( 89.14) | 133 ( 85.26) | 64 ( 98.46) |  |
| Sedative | No | 73 ( 33.03) | 70 ( 44.87) | 3 ( 4.62) | <0.001 |
|  | Yes | 148 ( 66.97) | 86 ( 55.13) | 62 ( 95.38) |  |
| Analgesic | No | 2 ( 0.90) | 2 ( 1.28) | 0 ( 0.00) | 0.891 |
|  | Yes | 219 ( 99.10) | 154 ( 98.72) | 65 (100.00) |  |
| Hypertension | No | 92 ( 41.63) | 63 ( 40.38) | 29 ( 44.62) | 0.666 |
|  | Yes | 129 ( 58.37) | 93 ( 59.62) | 36 ( 55.38) |  |
| HF | No | 196 ( 88.69) | 140 ( 89.74) | 56 ( 86.15) | 0.593 |
|  | Yes | 25 ( 11.31) | 16 ( 10.26) | 9 ( 13.85) |  |
| Diabetes | No | 215 ( 97.29) | 152 ( 97.44) | 63 ( 96.92) | 1 |
|  | Yes | 6 ( 2.71) | 4 ( 2.56) | 2 ( 3.08) |  |
| CKD | No | 211 ( 95.48) | 152 ( 97.44) | 59 ( 90.77) | 0.069 |
|  | Yes | 10 ( 4.52) | 4 ( 2.56) | 6 ( 9.23) |  |
| Pneumonia | No | 175 ( 79.19) | 141 ( 90.38) | 34 ( 52.31) | <0.001 |
|  | Yes | 46 ( 20.81) | 15 ( 9.62) | 31 ( 47.69) |  |
| Coagulopathy | No | 179 ( 81.00) | 130 ( 83.33) | 49 ( 75.38) | 0.236 |
|  | Yes | 42 ( 19.00) | 26 ( 16.67) | 16 ( 24.62) |  |
| Age(years)(median [IQR]) |  | 69.00 [57.00, 79.00] | 71.00 [59.00, 80.00] | 65.00 [55.00, 74.00] | 0.04 |
| Weight(kg) (median [IQR]) |  | 77.20 [68.00, 89.80] | 77.30 [68.42, 88.00] | 75.00 [66.80, 97.00] | 0.925 |
| SOFA (median [IQR]) |  | 3.00 [2.00, 4.00] | 2.00 [1.00, 4.00] | 4.00 [2.00, 5.00] | <0.001 |
| GCS (median [IQR]) |  | 14.00 [11.00, 15.00] | 14.00 [12.00, 15.00] | 14.00 [8.00, 15.00] | 0.956 |
| HR(bpm)(median [IQR]) |  | 78.00 [69.00, 89.00] | 76.50 [67.75, 87.25] | 81.00 [71.00, 90.00] | 0.129 |
| SBP(mmHg)(median [IQR]) |  | 134.00 [117.00, 145.00] | 133.00 [118.75, 146.00] | 135.00 [110.00, 144.00] | 0.408 |
| DBP(mmHg)(median [IQR]) |  | 69.00 [59.00, 80.00] | 69.00 [59.00, 79.25] | 69.00 [60.00, 81.00] | 0.887 |
| MAP(mmHg)(median [IQR]) |  | 85.00 [73.00, 95.00] | 84.00 [73.00, 96.00] | 86.00 [74.00, 93.00] | 0.788 |
| SpO2(%)(median [IQR]) |  | 99.00 [97.00, 100.00] | 98.50 [96.00, 100.00] | 100.00 [98.00, 100.00] | <0.001 |
| Glucose(mmol/L)(median [IQR]) |  | 7.11 [6.11, 8.67] | 6.89 [5.83, 8.33] | 7.83 [6.72, 9.50] | 0.005 |
| Chloride(mmol/L)(median [IQR]) |  | 105.00 [102.00, 108.00] | 105.00 [102.00, 108.00] | 105.00 [102.00, 108.00] | 0.755 |
| Potassium(mmol/L)(median [IQR]) |  | 3.90 [3.60, 4.20] | 4.00 [3.60, 4.20] | 3.90 [3.50, 4.20] | 0.167 |
| BUN(mg/dL)(median [IQR]) |  | 15.00 [11.00, 19.00] | 15.00 [11.00, 19.00] | 16.00 [12.00, 19.00] | 0.464 |
| Hematocrit(%)(median [IQR]) |  | 33.92 (5.58) | 34.54 (5.41) | 32.41 (5.74) | 0.009 |
| Hemoglobin(g/dL)(mean (SD)) |  | 11.53 (2.01) | 11.75 (2.00) | 11.01 (1.95) | 0.011 |
| RDW(%)(median [IQR]) |  | 13.90 [13.30, 15.10] | 13.90 [13.30, 14.72] | 14.30 [13.50, 15.50] | 0.067 |
| Platelet(×10⁹/L)(median [IQR]) |  | 209.00 [159.00, 262.00] | 217.50 [165.50, 263.50] | 194.00 [143.00, 250.00] | 0.168 |
| WBC(×10⁹/L)(median [IQR]) |  | 8.90 [6.70, 11.40] | 8.70 [6.68, 10.62] | 10.30 [7.30, 13.10] | 0.008 |
| RBC(×10¹²/L)(mean (SD)) |  | 3.83 (0.71) | 3.90 (0.72) | 3.65 (0.65) | 0.016 |
| Bicarbonate(mmol/L)(median [IQR]) |  | 25.00 [23.00, 27.00] | 25.00 [23.00, 27.00] | 24.00 [22.00, 27.00] | 0.082 |
| Calcium(mg/dL)(median [IQR]) |  | 8.60 [8.20, 9.10] | 8.80 [8.30, 9.20] | 8.50 [8.00, 8.80] | 0.002 |
| MCHC(g/dL)(median [IQR]) |  | 33.98 (1.55) | 33.97 (1.58) | 34.00 (1.48) | 0.896 |
| MCV(fL)(median [IQR]) |  | 90.00 [85.00, 94.00] | 89.00 [85.75, 93.00] | 90.00 [84.00, 94.00] | 0.869 |

**Table S4. Baseline Characteristics of ICU Patients Undergoing Surgical Evacuation of Intracranial Hematoma in the eICU Database According to Postoperative Sepsis Status**

| **Characteristics** | **level** | **Overall(n=302)** | **Nonsepsis(n=225)** | **Sepsis(n=77)** | ***p*** |
| --- | --- | --- | --- | --- | --- |
| Sex | Female | 110 (36.42) | 86 ( 38.22) | 24 (31.17) | 0.331 |
|  | Male | 192 (63.58) | 139 ( 61.78) | 53 (68.83) |  |
| MV | No | 193 (63.91) | 177 ( 78.67) | 16 (20.78) | <0.001 |
|  | Yes | 109 (36.09) | 48 ( 21.33) | 61 (79.22) |  |
| Vasopressor | No | 248 (82.12) | 190 ( 84.44) | 58 (75.32) | 0.103 |
|  | Yes | 54 (17.88) | 35 ( 15.56) | 19 (24.68) |  |
| CRRT | No | 298 (98.68) | 224 ( 99.56) | 74 (96.10) | 0.087 |
|  | Yes | 4 ( 1.32) | 1 ( 0.44) | 3 ( 3.90) |  |
| Mannitol | No | 290 (96.03) | 220 ( 97.78) | 70 (90.91) | 0.02 |
|  | Yes | 12 ( 3.97) | 5 ( 2.22) | 7 ( 9.09) |  |
| Aspirin | No | 292 (96.69) | 218 ( 96.89) | 74 (96.10) | 1 |
|  | Yes | 10 ( 3.31) | 7 ( 3.11) | 3 ( 3.90) |  |
| Heparin | No | 243 (80.46) | 186 ( 82.67) | 57 (74.03) | 0.138 |
|  | Yes | 59 (19.54) | 39 ( 17.33) | 20 (25.97) |  |
| Sedative | No | 156 (51.66) | 128 ( 56.89) | 28 (36.36) | 0.003 |
|  | Yes | 146 (48.34) | 97 ( 43.11) | 49 (63.64) |  |
| Analgesic | No | 65 (21.52) | 50 ( 22.22) | 15 (19.48) | 0.73 |
|  | Yes | 237 (78.48) | 175 ( 77.78) | 62 (80.52) |  |
| Hypertension | No | 217 (71.85) | 180 ( 80.00) | 37 (48.05) | <0.001 |
|  | Yes | 85 (28.15) | 45 ( 20.00) | 40 (51.95) |  |
| HF | No | 295 (97.68) | 225 (100.00) | 70 (90.91) | <0.001 |
|  | Yes | 7 ( 2.32) | 0 ( 0.00) | 7 ( 9.09) |  |
| Diabetes | No | 280 (92.72) | 213 ( 94.67) | 67 (87.01) | 0.048 |
|  | Yes | 22 ( 7.28) | 12 ( 5.33) | 10 (12.99) |  |
| CKD | No | 288 (95.36) | 218 ( 96.89) | 70 (90.91) | 0.066 |
|  | Yes | 14 ( 4.64) | 7 ( 3.11) | 7 ( 9.09) |  |
| Pneumonia | No | 287 (95.03) | 222 ( 98.67) | 65 (84.42) | <0.001 |
|  | Yes | 15 ( 4.97) | 3 ( 1.33) | 12 (15.58) |  |
| Coagulopathy | No | 285 (94.37) | 217 ( 96.44) | 68 (88.31) | 0.017 |
|  | Yes | 17 ( 5.63) | 8 ( 3.56) | 9 (11.69) |  |
| Age(years)(median [IQR]) |  | 71.00 [57.00, 81.00] | 71.00 [58.00, 81.00] | 71.00 [54.00, 81.00] | 0.863 |
| Weight(kg) (median [IQR]) |  | 74.90 [66.03, 88.40] | 75.00 [66.50, 88.70] | 73.80 [65.10, 86.40] | 0.909 |
| SOFA (median [IQR]) |  | 3.00 [1.00, 5.00] | 2.00 [1.00, 4.00] | 5.00 [3.00, 6.00] | <0.001 |
| GCS (median [IQR]) |  | 14.00 [10.00, 15.00] | 14.00 [12.00, 15.00] | 10.00 [7.00, 12.00] | <0.001 |
| HR(bpm)(median [IQR]) |  | 78.00 [68.00, 88.75] | 78.00 [68.00, 87.00] | 79.00 [66.00, 92.00] | 0.674 |
| SBP(mmHg)(median [IQR]) |  | 134.64 (21.22) | 134.55 (19.65) | 134.91 (25.39) | 0.899 |
| DBP(mmHg)(median [IQR]) |  | 71.00 [60.00, 79.00] | 72.00 [62.00, 80.00] | 66.00 [56.00, 78.00] | 0.057 |
| MAP(mmHg)(median [IQR]) |  | 88.00 [78.00, 97.00] | 90.00 [80.00, 98.00] | 84.00 [74.00, 94.00] | 0.026 |
| SpO2(%)(median [IQR]) |  | 99.00 [97.00, 100.00] | 98.00 [97.00, 100.00] | 100.00 [97.00, 100.00] | 0.058 |
| Glucose(mmol/L)(median [IQR]) |  | 8.47 [7.00, 10.54] | 8.06 [6.72, 9.72] | 9.89 [8.00, 12.33] | <0.001 |
| Chloride(mmol/L)(median [IQR]) |  | 109.00 [105.00, 113.00] | 108.00 [105.00, 111.00] | 115.00 [109.00, 120.00] | <0.001 |
| Potassium(mmol/L)(median [IQR]) |  | 4.30 [4.00, 4.60] | 4.20 [4.00, 4.60] | 4.40 [4.10, 4.80] | 0.001 |
| BUN(mg/dL)(median [IQR]) |  | 20.00 [14.00, 29.75] | 18.00 [13.00, 25.00] | 28.00 [20.00, 37.00] | <0.001 |
| Hematocrit(%)(median [IQR]) |  | 35.70 [32.15, 39.92] | 36.10 [33.00, 40.20] | 33.80 [31.10, 38.80] | 0.007 |
| Hemoglobin(g/dL)(mean (SD)) |  | 11.90 [10.70, 13.30] | 11.90 [10.80, 13.50] | 11.40 [10.10, 13.10] | 0.03 |
| RDW(%)(median [IQR]) |  | 14.50 [13.60, 16.00] | 14.40 [13.30, 15.90] | 15.00 [14.40, 16.30] | <0.001 |
| Platelet(×10⁹/L)(median [IQR]) |  | 261.00 [205.00, 337.50] | 249.00 [206.00, 326.00] | 302.00 [201.00, 400.00] | 0.035 |
| WBC(×10⁹/L)(median [IQR]) |  | 12.80 [9.80, 16.38] | 12.00 [9.20, 15.80] | 15.50 [12.80, 17.93] | <0.001 |
| RBC(×10¹²/L)(mean (SD)) |  | 3.98 [3.52, 4.42] | 4.08 [3.59, 4.50] | 3.66 [3.37, 4.26] | 0.002 |
| Bicarbonate(mmol/L)(median [IQR]) |  | 27.00 [25.00, 29.00] | 27.00 [25.00, 29.00] | 27.00 [25.00, 30.00] | 0.544 |
| Calcium(mg/dL)(median [IQR]) |  | 8.90 [8.60, 9.30] | 8.90 [8.60, 9.20] | 8.80 [8.60, 9.30] | 0.77 |
| MCHC(g/dL)(median [IQR]) |  | 34.00 [33.10, 34.70] | 33.90 [33.00, 34.60] | 34.00 [33.40, 35.00] | 0.042 |
| MCV(fL)(median [IQR]) |  | 92.00 [87.93, 95.42] | 92.00 [87.00, 95.00] | 93.10 [90.30, 97.00] | 0.023 |

**Table S5. Baseline Characteristics of ICU Patients Undergoing Surgical Evacuation of Intracranial Hematoma in the Jiangsu Provincial Hospital of Integrated Traditional Chinese and Western Medicine Cohort According to Postoperative Sepsis Status**

| **Characteristics** | **level** | **Overall(n=180)** | **Nonsepsis(n=123)** | **Sepsis(n=57)** | ***p*** |
| --- | --- | --- | --- | --- | --- |
| Sex | Female | 58 ( 32.22) | 43 ( 34.96) | 15 ( 26.32) | 0.326 |
|  | Male | 122 ( 67.78) | 80 ( 65.04) | 42 ( 73.68) |  |
| MV | No | 14 ( 7.78) | 12 ( 9.76) | 2 ( 3.51) | 0.247 |
|  | Yes | 166 ( 92.22) | 111 ( 90.24) | 55 ( 96.49) |  |
| Vasopressor | No | 86 ( 47.78) | 82 ( 66.67) | 4 ( 7.02) | <0.001 |
|  | Yes | 94 ( 52.22) | 41 ( 33.33) | 53 ( 92.98) |  |
| CRRT | No | 179 ( 99.44) | 122 ( 99.19) | 57 (100.00) | 1 |
|  | Yes | 1 ( 0.56) | 1 ( 0.81) | 0 ( 0.00) |  |
| Mannitol | No | 62 ( 34.44) | 43 ( 34.96) | 19 ( 33.33) | 0.964 |
|  | Yes | 118 ( 65.56) | 80 ( 65.04) | 38 ( 66.67) |  |
| Aspirin | No | 179 ( 99.44) | 123 (100.00) | 56 ( 98.25) | 0.693 |
|  | Yes | 1 ( 0.56) | 0 ( 0.00) | 1 ( 1.75) |  |
| Heparin | No | 177 ( 98.33) | 120 ( 97.56) | 57 (100.00) | 0.573 |
|  | Yes | 3 ( 1.67) | 3 ( 2.44) | 0 ( 0.00) |  |
| Sedative | No | 16 ( 8.89) | 10 ( 8.13) | 6 ( 10.53) | 0.807 |
|  | Yes | 164 ( 91.11) | 113 ( 91.87) | 51 ( 89.47) |  |
| Analgesic | No | 39 ( 21.67) | 29 ( 23.58) | 10 ( 17.54) | 0.472 |
|  | Yes | 141 ( 78.33) | 94 ( 76.42) | 47 ( 82.46) |  |
| Hypertension | No | 86 ( 47.78) | 57 ( 46.34) | 29 ( 50.88) | 0.685 |
|  | Yes | 94 ( 52.22) | 66 ( 53.66) | 28 ( 49.12) |  |
| HF | No | 174 ( 96.67) | 122 ( 99.19) | 52 ( 91.23) | 0.02 |
|  | Yes | 6 ( 3.33) | 1 ( 0.81) | 5 ( 8.77) |  |
| Diabetes | No | 139 ( 77.22) | 92 ( 74.80) | 47 ( 82.46) | 0.343 |
|  | Yes | 41 ( 22.78) | 31 ( 25.20) | 10 ( 17.54) |  |
| CKD | No | 173 ( 96.11) | 121 ( 98.37) | 52 ( 91.23) | 0.058 |
|  | Yes | 7 ( 3.89) | 2 ( 1.63) | 5 ( 8.77) |  |
| Pneumonia | No | 127 ( 70.56) | 90 ( 73.17) | 37 ( 64.91) | 0.34 |
|  | Yes | 53 ( 29.44) | 33 ( 26.83) | 20 ( 35.09) |  |
| Coagulopathy | No | 180 (100.00) | 123 (100.00) | 57 (100.00) |  |
| Age(years)(median [IQR]) |  | 58.22 (14.80) | 57.72 (14.55) | 59.28 (15.40) | 0.513 |
| Weight(kg) (median [IQR]) |  | 70.00 [62.00, 80.00] | 69.00 [60.00, 80.00] | 71.00 [65.00, 82.00] | 0.092 |
| SOFA (median [IQR]) |  | 7.00 [6.00, 10.00] | 6.00 [5.00, 7.00] | 11.00 [9.00, 12.00] | <0.001 |
| GCS (median [IQR]) |  | 3.00 [2.00, 8.00] | 4.00 [2.00, 11.00] | 3.00 [2.00, 4.00] | 0.002 |
| HR(bpm)(median [IQR]) |  | 80.00 [69.75, 90.00] | 78.00 [66.00, 87.00] | 88.00 [76.00, 99.00] | <0.001 |
| SBP(mmHg)(median [IQR]) |  | 136.93 (30.66) | 137.49 (29.59) | 135.72 (33.11) | 0.72 |
| DBP(mmHg)(median [IQR]) |  | 71.64 (17.05) | 72.28 (17.05) | 70.25 (17.12) | 0.457 |
| MAP(mmHg)(median [IQR]) |  | 93.36 (18.71) | 94.01 (18.05) | 91.96 (20.14) | 0.495 |
| SpO2(%)(median [IQR]) |  | 100.00 [99.00, 100.00] | 100.00 [99.00, 100.00] | 100.00 [99.00, 100.00] | 0.512 |
| Glucose(mmol/L)(median [IQR]) |  | 9.20 [7.19, 11.31] | 8.76 [6.98, 11.16] | 10.20 [8.29, 12.05] | 0.024 |
| Chloride(mmol/L)(median [IQR]) |  | 107.40 [104.30, 111.00] | 107.00 [104.00, 110.55] | 108.10 [105.20, 111.70] | 0.049 |
| Potassium(mmol/L)(median [IQR]) |  | 3.78 [3.43, 4.16] | 3.74 [3.41, 4.10] | 3.88 [3.47, 4.37] | 0.036 |
| BUN(mg/dL)(median [IQR]) |  | 17.40 [13.42, 24.86] | 16.35 [12.91, 21.38] | 23.24 [15.12, 33.01] | <0.001 |
| Hematocrit(%)(median [IQR]) |  | 32.57 (7.36) | 32.82 (6.94) | 32.04 (8.23) | 0.513 |
| Hemoglobin(g/dL)(mean (SD)) |  | 10.85 (2.49) | 11.01 (2.37) | 10.51 (2.73) | 0.205 |
| RDW(%)(median [IQR]) |  | 13.20 [12.60, 13.90] | 13.10 [12.50, 13.85] | 13.40 [12.90, 13.90] | 0.06 |
| Platelet(×10⁹/L)(median [IQR]) |  | 157.00 [111.00, 196.50] | 166.00 [124.00, 199.00] | 133.00 [92.00, 186.00] | 0.008 |
| WBC(×10⁹/L)(median [IQR]) |  | 12.31 [9.52, 15.98] | 11.58 [9.20, 15.50] | 13.69 [10.00, 17.27] | 0.151 |
| RBC(×10¹²/L)(mean (SD)) |  | 3.47 (0.81) | 3.52 (0.76) | 3.36 (0.92) | 0.226 |
| Bicarbonate(mmol/L)(median [IQR]) |  | 22.74 (3.39) | 22.77 (3.30) | 22.66 (3.60) | 0.838 |
| Calcium(mg/dL)(median [IQR]) |  | 8.10 [7.65, 8.46] | 8.18 [7.82, 8.46] | 7.90 [7.37, 8.42] | 0.075 |
| MCHC(g/dL)(median [IQR]) |  | 33.70 (0.92) | 33.72 (0.90) | 33.66 (0.97) | 0.704 |
| MCV(fL)(median [IQR]) |  | 92.45 [89.40, 94.50] | 92.70 [89.65, 94.95] | 91.80 [89.40, 94.00] | 0.217 |

**Table S6. DeLong test results comparing XGBoost with selected high-performing models in the internal test and MIMIC-III external validation cohorts**

| **cohort** | **n** | **model1** | **model2** | **auc_model1** | **auc_model2** | ***z*** | ***p*** | **features_used** |
| --- | --- | --- | --- | --- | --- | --- | --- | --- |
| Test set | 307 | XGBoost | RandomForest | 0.7939728353140917 | 0.8017826825127334 | -1.338694863 | 0.18067002928507891 | WBC,SOFA,SpO2,  Glucose,Chloride,  Pneumonia,Aspirin,  Mannitol,  Vasopressor,MV,  RBC,Sedative,GCS |
| MIMIC-III external validation set | 221 | XGBoost | RandomForest | 0.8199211045364891 | 0.8273175542406311 | -0.947289912 | 0.34349106986937294 | WBC,SOFA,SpO2,  Glucose,Chloride,  Pneumonia,Aspirin,  Mannitol,  Vasopressor,MV,  RBC,Sedative,GCS |
| MIMIC-III external validation set | 221 | XGBoost | SVM | 0.8199211045364891 | 0.821301775147929 | -0.072351308 | 0.9423223341077287 | WBC,SOFA,SpO2,  Glucose,Chloride,  Pneumonia,Aspirin,  Mannitol,  Vasopressor,MV,  RBC,Sedative,GCS |
| MIMIC-III external validation set | 221 | XGBoost | ANN | 0.8199211045364891 | 0.8298816568047337 | -0.77070779 | 0.44088015345700615 | WBC,SOFA,SpO2,  Glucose,Chloride,  Pneumonia,Aspirin,  Mannitol,  Vasopressor,MV,  RBC,Sedative,GCS |

**Table S7. Comparison of discriminative performance between the original and SOFA-free machine learning models across cohorts**

| **Cohort** | **Model** | **Original AUC (95% CI)** | **SOFA-free AUC (95% CI)** | **ΔAUC** | **DeLong**  ***P-*value** |
| --- | --- | --- | --- | --- | --- |
| MIMIC-IV Train | Decision Tree | 0.809 (0.780–0.838) | 0.815 (0.782–0.845) | 0.006 | — |
|  | Random Forest | 0.934 (0.918–0.949) | 0.929 (0.912–0.944) | -0.005 | — |
|  | XGBoost | 0.889 (0.867–0.910) | 0.879 (0.855–0.901) | -0.011 | — |
|  | LightGBM | 0.967 (0.956–0.977) | 0.957 (0.944–0.969) | -0.010 | — |
|  | SVM | 0.824 (0.795–0.852) | 0.822 (0.790–0.850) | -0.002 | — |
|  | ANN | 0.856 (0.829–0.879) | 0.810 (0.780–0.839) | -0.046 | — |
| MIMIC-IV Test | Decision Tree | 0.729 (0.671–0.781) | 0.676 (0.618–0.731) | -0.054 | 0.058 |
|  | Random Forest | 0.802 (0.747–0.844) | 0.771 (0.716–0.816) | -0.031 | 0.001 |
|  | XGBoost | 0.794 (0.738–0.838) | 0.757 (0.699–0.805) | -0.037 | 0.001 |
|  | LightGBM | 0.750 (0.687–0.801) | 0.739 (0.679–0.791) | -0.011 | 0.401 |
|  | SVM | 0.787 (0.732–0.833) | 0.761 (0.703–0.809) | -0.026 | 0.003 |
|  | ANN | 0.790 (0.736–0.834) | 0.762 (0.705–0.809) | -0.028 | 0.017 |
| MIMIC-III | Decision Tree | 0.740 (0.668–0.806) | 0.706 (0.633–0.771) | -0.034 | 0.339 |
|  | Random Forest | 0.827 (0.766–0.883) | 0.838 (0.781–0.890) | 0.010 | 0.342 |
|  | XGBoost | 0.820 (0.753–0.880) | 0.811 (0.749–0.865) | -0.009 | 0.458 |
|  | LightGBM | 0.793 (0.718–0.856) | 0.794 (0.728–0.851) | 0.000 | 0.984 |
|  | SVM | 0.821 (0.761–0.877) | 0.814 (0.753–0.870) | -0.008 | 0.387 |
|  | ANN | 0.830 (0.767–0.883) | 0.821 (0.764–0.875) | -0.009 | 0.521 |
| eICU | Decision Tree | 0.741 (0.685–0.795) | 0.688 (0.627–0.749) | -0.052 | 0.054 |
|  | Random Forest | 0.824 (0.775–0.874) | 0.814 (0.766–0.863) | -0.010 | 0.341 |
|  | XGBoost | 0.837 (0.788–0.886) | 0.823 (0.777–0.871) | -0.014 | 0.226 |
|  | LightGBM | 0.824 (0.774–0.871) | 0.797 (0.746–0.849) | -0.027 | 0.029 |
|  | SVM | 0.815 (0.761–0.869) | 0.806 (0.753–0.858) | -0.009 | 0.374 |
|  | ANN | 0.830 (0.778–0.879) | 0.825 (0.775–0.873) | -0.005 | 0.724 |
| JPHITCWM | Decision Tree | 0.782 (0.715–0.842) | 0.686 (0.603–0.764) | -0.096 | 0.023 |
|  | Random Forest | 0.688 (0.608–0.768) | 0.798 (0.725–0.863) | 0.109 | <0.001 |
|  | XGBoost | 0.796 (0.724–0.862) | 0.772 (0.699–0.844) | -0.024 | 0.081 |
|  | LightGBM | 0.705 (0.626–0.783) | 0.711 (0.626–0.790) | 0.007 | 0.748 |
|  | SVM | 0.747 (0.668–0.820) | 0.705 (0.620–0.786) | -0.042 | 0.029 |
|  | ANN | 0.778 (0.713–0.845) | 0.735 (0.658–0.806) | -0.043 | 0.246 |

**Table S8. Performance of the SOFA-free machine learning models across the development and validation cohorts**

| **Data set** | **Model** | **AUC** | **AUC 95% CI Lower** | **AUC 95% CI Upper** | **Accuracy** | **Precision** | **Sensitivity** | **Specificity** | **F1 Score** | **Kappa** | **Youden's J** | **PPV** | **NPV** |
| --- | --- | --- | --- | --- | --- | --- | --- | --- | --- | --- | --- | --- | --- |
| MIMIC-IV Training | Decision Tree | 0.815 | 0.782 | 0.845 | 0.747 | 0.785 | 0.686 | 0.809 | 0.732 | 0.494 | 0.495 | 0.785 | 0.716 |
|  | Random Forest | 0.929 | 0.912 | 0.944 | 0.848 | 0.826 | 0.887 | 0.809 | 0.855 | 0.697 | 0.696 | 0.826 | 0.875 |
|  | XGBoost | 0.879 | 0.855 | 0.901 | 0.789 | 0.778 | 0.813 | 0.764 | 0.795 | 0.577 | 0.577 | 0.778 | 0.800 |
|  | LightGBM | 0.957 | 0.944 | 0.969 | 0.880 | 0.886 | 0.876 | 0.885 | 0.881 | 0.761 | 0.761 | 0.886 | 0.875 |
|  | SVM | 0.822 | 0.790 | 0.850 | 0.744 | 0.752 | 0.736 | 0.753 | 0.744 | 0.488 | 0.488 | 0.752 | 0.736 |
|  | ANN | 0.810 | 0.780 | 0.839 | 0.737 | 0.728 | 0.766 | 0.708 | 0.746 | 0.474 | 0.474 | 0.728 | 0.748 |
| MIMIC-IV Test | Decision Tree | 0.676 | 0.618 | 0.731 | 0.625 | 0.672 | 0.503 | 0.750 | 0.576 | 0.253 | 0.253 | 0.672 | 0.597 |
|  | Random Forest | 0.771 | 0.716 | 0.816 | 0.681 | 0.691 | 0.665 | 0.697 | 0.678 | 0.362 | 0.362 | 0.691 | 0.671 |
|  | XGBoost | 0.757 | 0.699 | 0.805 | 0.661 | 0.689 | 0.600 | 0.724 | 0.641 | 0.323 | 0.324 | 0.689 | 0.640 |
|  | LightGBM | 0.739 | 0.679 | 0.791 | 0.658 | 0.662 | 0.658 | 0.658 | 0.660 | 0.316 | 0.316 | 0.662 | 0.654 |
|  | SVM | 0.761 | 0.703 | 0.809 | 0.691 | 0.738 | 0.600 | 0.783 | 0.662 | 0.382 | 0.383 | 0.738 | 0.657 |
|  | ANN | 0.762 | 0.705 | 0.809 | 0.687 | 0.715 | 0.632 | 0.743 | 0.671 | 0.375 | 0.376 | 0.715 | 0.665 |
| MIMIC-III | Decision Tree | 0.706 | 0.633 | 0.771 | 0.652 | 0.441 | 0.692 | 0.635 | 0.539 | 0.280 | 0.327 | 0.441 | 0.832 |
|  | Random Forest | 0.838 | 0.781 | 0.890 | 0.647 | 0.449 | 0.877 | 0.551 | 0.594 | 0.335 | 0.428 | 0.449 | 0.915 |
|  | XGBoost | 0.811 | 0.749 | 0.865 | 0.679 | 0.475 | 0.892 | 0.590 | 0.620 | 0.384 | 0.482 | 0.475 | 0.929 |
|  | LightGBM | 0.794 | 0.728 | 0.851 | 0.670 | 0.467 | 0.877 | 0.583 | 0.610 | 0.367 | 0.460 | 0.467 | 0.919 |
|  | SVM | 0.814 | 0.753 | 0.870 | 0.706 | 0.500 | 0.846 | 0.647 | 0.629 | 0.411 | 0.494 | 0.500 | 0.910 |
|  | ANN | 0.821 | 0.764 | 0.875 | 0.674 | 0.470 | 0.831 | 0.609 | 0.600 | 0.359 | 0.440 | 0.470 | 0.896 |
| eICU | Decision Tree | 0.688 | 0.627 | 0.749 | 0.669 | 0.394 | 0.558 | 0.707 | 0.462 | 0.233 | 0.265 | 0.394 | 0.824 |
|  | Random Forest | 0.814 | 0.766 | 0.863 | 0.679 | 0.438 | 0.909 | 0.600 | 0.591 | 0.376 | 0.509 | 0.438 | 0.951 |
|  | XGBoost | 0.823 | 0.777 | 0.871 | 0.669 | 0.426 | 0.857 | 0.604 | 0.569 | 0.346 | 0.462 | 0.426 | 0.925 |
|  | LightGBM | 0.797 | 0.746 | 0.849 | 0.666 | 0.423 | 0.857 | 0.600 | 0.567 | 0.342 | 0.457 | 0.423 | 0.925 |
|  | SVM | 0.806 | 0.753 | 0.858 | 0.646 | 0.405 | 0.831 | 0.582 | 0.545 | 0.307 | 0.413 | 0.405 | 0.910 |
|  | ANN | 0.825 | 0.775 | 0.873 | 0.679 | 0.432 | 0.831 | 0.627 | 0.569 | 0.351 | 0.458 | 0.432 | 0.916 |
| JPHITCWM | Decision Tree | 0.686 | 0.603 | 0.764 | 0.494 | 0.385 | 1.000 | 0.260 | 0.556 | 0.182 | 0.260 | 0.385 | 1.000 |
|  | Random Forest | 0.798 | 0.725 | 0.863 | 0.333 | 0.322 | 1.000 | 0.024 | 0.487 | 0.016 | 0.024 | 0.322 | 1.000 |
|  | XGBoost | 0.772 | 0.699 | 0.844 | 0.328 | 0.320 | 1.000 | 0.016 | 0.485 | 0.010 | 0.016 | 0.320 | 1.000 |
|  | LightGBM | 0.711 | 0.626 | 0.790 | 0.356 | 0.329 | 1.000 | 0.057 | 0.496 | 0.037 | 0.057 | 0.329 | 1.000 |
|  | SVM | 0.705 | 0.620 | 0.786 | 0.339 | 0.324 | 1.000 | 0.033 | 0.489 | 0.021 | 0.033 | 0.324 | 1.000 |
|  | ANN | 0.735 | 0.658 | 0.806 | 0.333 | 0.322 | 1.000 | 0.024 | 0.487 | 0.016 | 0.024 | 0.322 | 1.000 |

**Table S9. Selected hyperparameters for the six machine learning models.**

| **Model** | **Selected hyperparameters** |
| --- | --- |
| Decision Tree | ccp_alpha=0.0005; max_depth=8; max_features=sqrt; min_samples_split=50 |
| Random Forest | n_estimators=400; max_features=sqrt |
| XGBoost | learning_rate=0.021; max_depth=4; n_estimators=300; subsample=1.0 |
| LightGBM | colsample_bytree=0.9; learning_rate=0.03; n_estimators=400; num_leaves=31; subsample=0.7 |
| SVM | C=100; degree=2; gamma=0.0003; kernel=rbf |
| ANN | activation=relu; hidden_layer_sizes=(100,) |

**Figure S1. Missing data rates of candidate variables in the MIMIC-IV cohort**

**Figure S2. Missing data rates of candidate variables in the MIMIC-III cohort**

**Figure S3. Missing data rates of candidate variables in the eICU cohort**

**Figure S4. Missing data rates of candidate variables in the JPHITCWM cohort**

**Figure S5. Calibration curves of the six machine learning models in the training cohort**

**Figure S6. Calibration curves of the six machine learning models in the internal test cohort**

**Figure S7. Calibration curves of the six machine learning models in the MIMIC-III external validation cohort**

**Figure S8. Calibration curves of the six machine learning models in the eICU external validation cohort**

**Figure S9. Calibration curves of the six machine learning models in the JPHITCWM external validation cohort**

**Figure S10. Decision curve analysis of the six machine learning models in the training cohort**

**Figure S11. Decision curve analysis of the six machine learning models in the internal test cohort**

**Figure S12. Decision curve analysis of the six machine learning models in the MIMIC-III external validation cohort**

**Figure S13. Decision curve analysis of the six machine learning models in the eICU external validation cohort**

**Figure S14. Decision curve analysis of the six machine learning models in the JPHITCWM external validation cohort**

**Figure. S15 LIME interpretation of the XGBoost model.**
(A) Ranked feature contributions for a representative high-risk case: bar length indicates the magnitude of each feature contribution, and colors denote the direction of effect. The top ten contributing predictors were SOFA score, mannitol use, vasopressor use, pneumonia, mechanical ventilation, aspirin use, sedative use, SpO₂, chloride, and glucose. (B) Example case prediction: the predicted probability of high risk was 0.91, whereas the predicted probability of low risk was 0.09. SOFA score between 2 and 3, vasopressor use, pneumonia, mechanical ventilation, aspirin use, sedative use, higher SpO₂, and higher chloride contributed positively to the high-risk prediction, whereas no mannitol use and lower glucose contributed in the opposite direction.

Note: SOFA score category: 2.00 < SOFA ≤ 3.00; SpO₂ category: 99.00 < SpO₂ ≤ 100.00; chloride category: chloride >107.00; glucose category: glucose ≤5.83. Binary variables were coded as yes/no, including vasopressor use, pneumonia, mechanical ventilation, aspirin use, sedative use, and mannitol use.

**Figure S16. Confusion matrices of six machine learning models in the JPHITCWM external validation cohort.**

**Figure S17. Original Chinese ethics approval document.**

**Figure S18. English translation of the ethics approval document.**

**Figure S19. ROC curves of the six SOFA-free machine learning models in the MIMIC-IV training set.**

**Figure S20. ROC curves of the six SOFA-free machine learning models in the MIMIC-IV internal test set.**

**Figure S21. ROC curves of the six SOFA-free machine learning models in the MIMIC-III cohort.**

**Figure S22. ROC curves of the six SOFA-free machine learning models in the eICU cohort.**

**Figure S23. ROC curves of the six SOFA-free machine learning models in the JPHITCWM cohort.**

**Figure S24. Global SHAP feature importance of the SOFA-free XGBoost model.**

**Figure S25. Spearman correlation matrix of the final predictors in the MIMIC-IV training set.**
